# Structured Treatment Interruptions and Low Doses of IL-2 in Patients with Primary HIV Infection. Inflammatory, Virological and Immunological Outcomes

**DOI:** 10.1371/journal.pone.0131651

**Published:** 2015-07-17

**Authors:** Omar Sued, Juan Ambrosioni, David Nicolás, Christian Manzardo, Fernando Agüero, Xavier Claramonte, Montserrat Plana, Montserrat Tuset, Tomás Pumarola, Teresa Gallart, José María Gatell, José María Miró

**Affiliations:** 1 Infectious Diseases Service, Hospital Clínic–IDIBAPS, University of Barcelona, Barcelona, Spain; 2 Pharmacy Service, Hospital Clínic–IDIBAPS, University of Barcelona, Barcelona, Spain; Rush University, UNITED STATES

## Abstract

**Background:**

Interventions during primary HIV infection (PHI) can modify the clinical course during the chronic phase. The long-term effect of structured treatment interruptions (STI) followed by low doses of interleukin-2 (IL-2) in treated PHI patients is unknown.

**Methods:**

Twelve PHI patients with viral load (VL) <20 copies/mL, CD4 cells >500 cells/mm3, and CD4/CD8 ratio >1, on antiretroviral therapy (ART) initiated within the first 90 days of infection and continued for at least 12 months were included. They underwent four STI and were then allocated (week 0 of the study) to ART alone or ART plus low doses of IL-2. ART was stopped once VL <20 copies/mL ('final stop'). Primary endpoints were VL<3000 copies/mL and CD4 cells >500 cells/mm3 at 48 weeks; secondary endpoints were immune activation, inflammatory markers until 48 weeks and the time before resuming ART (CD4 <350 cells/mm3 or AIDS) after ‘final stop’, compared between groups.

**Results:**

Ten out of 12 patients were males, median age was 35 years and the main risk was men-who-have-sex-with-men. Only one out of 12 patients (in the STI group) maintained VL<3000 copies/mL and CD4 cells >500 cells/mm3 without ART at 48 weeks. All other virological and immunological parameters were comparable between groups at week 0, 'final stop' and week 48. However, the proportion of CD8-CD38+ cells, tumor necrosis factor and srIL-2 were higher in the IL-2 group at 'final stop' and week 24. All these differences vanished during follow-up. At 5 years after the final stop 3 out of 6 patients in the IL-2 group and 6 out of 6 patients in the STI group have resumed ART (P = 0.19).

**Conclusions:**

STI and IL-2 failed to achieve virological control after ART interruption. STI were not deleterious in long-term follow-up, an important issue for eradication and functional cure trials.

**Trial Registration:**

ClinicalTrials.gov NCT02300623

## Introduction

Potent combination antiretroviral treatment (ART) during primary HIV infection (PHI) results in strong viral suppression rates, rapid recovery of CD4 T cells[1, 2], and a reduction of viral reservoirs[3, 4]. Moreover, ART in this clinical phase preserves HIV-specific T-cell helper (Th) and cytotoxic T lymphocyte (CTL) immune responses, improves surrogate markers of disease progression[5–7] [8] and reduces HIV transmission[9, 10]. The fact that ART during PHI preserves HIV-specific CD4 helper T-cell response[5–7] is remarkable, as this response is usually absent in chronically-infected patients, even in those receiving successful ART since the early asymptomatic phase of chronic infection[5, 11]. In fact, ART-treated PHI patients exhibit HIV-specific Th and CTL immune responses in a similar manner as long-term non-progressors (LTNP) or “elite controllers” that spontaneously control HIV replication[12, 13].

Soon after the introduction of potent ART, observational case reports identified patients receiving ART during PHI that controlled HIV replication after ART discontinuation and reported that this control was associated with strong HIV-specific cell-mediated immune responses[14, 15]. These cases lead to the hypothesis that brief exposures to the autologous virus during supervised or structured treatment interruptions (STI) in patients receiving ART since PHI might act by boosting HIV-specific immune responses, which could provide immune control of viral replication. STI were also evaluated in several clinical trials during chronic HIV infection[16–18]. The encouraging results obtained in a small clinical trial using three STIs in subjects receiving ART since PHI[7] and similar experimental findings in primates[19] further fueled interest in this approach and in other possible immunotherapeutic procedures to induce/boost HIV-specific immune responses[20–22]. Unfortunately, however, the viral control after STI in the mentioned trial with ART-treated PHI patients was found to have a limited durability[23]. Nevertheless, recently, two reports have renewed further interest in administering ART very early during PHI as a considerable proportion of patients can control HIV replication after ART cessation[24, 25]. The proportion of these post-ART controllers was considerably high compared to that of spontaneous “elite” controllers, and the former lack some genetic characteristics that are overrepresented among the latter, suggesting that the key for controlling HIV viremia was the early initiation of ART[24, 25].

In our trial, we used IL-2, one of the first cytokines discovered to promote T-cell growth[26], as an adjunctive immunotherapy aimed at favoring the clonal expansion of HIV-specific Th and CTL responses. We utilized daily s.c. ultra-low IL-2 dose, which has been demonstrated to be not only nontoxic and safe but also effective in stimulating immunoreactivity in patients with AIDS and with AIDS-related malignancies[27–29].

These considerations provided the rationale for this pilot clinical trial to evaluate the impact of STI during PHI with or without the addition of low-dose recombinant IL-2 to boost HIV-1 specific immune responses and achieve a control of HIV viremia. Although STI in chronic HIV infection has shown to increase the risk of opportunistic infection and death [30], it can also enhance host immune control of viral replication [17] and no deleterious effect was shown during PHI in a recently published study[31]. This issue is particularly important in the context of the trials for HIV-functional cure, in which ART interruptions need to be performed to test the control of viremia by the immune system. We report here the virological, immunological and inflammatory markers up to 48 weeks of follow-up and the time to require resuming ART (CD4<350/μl or AIDS events) in the long-term follow-up.

## Methods

### Patient population

**Fig 1:**
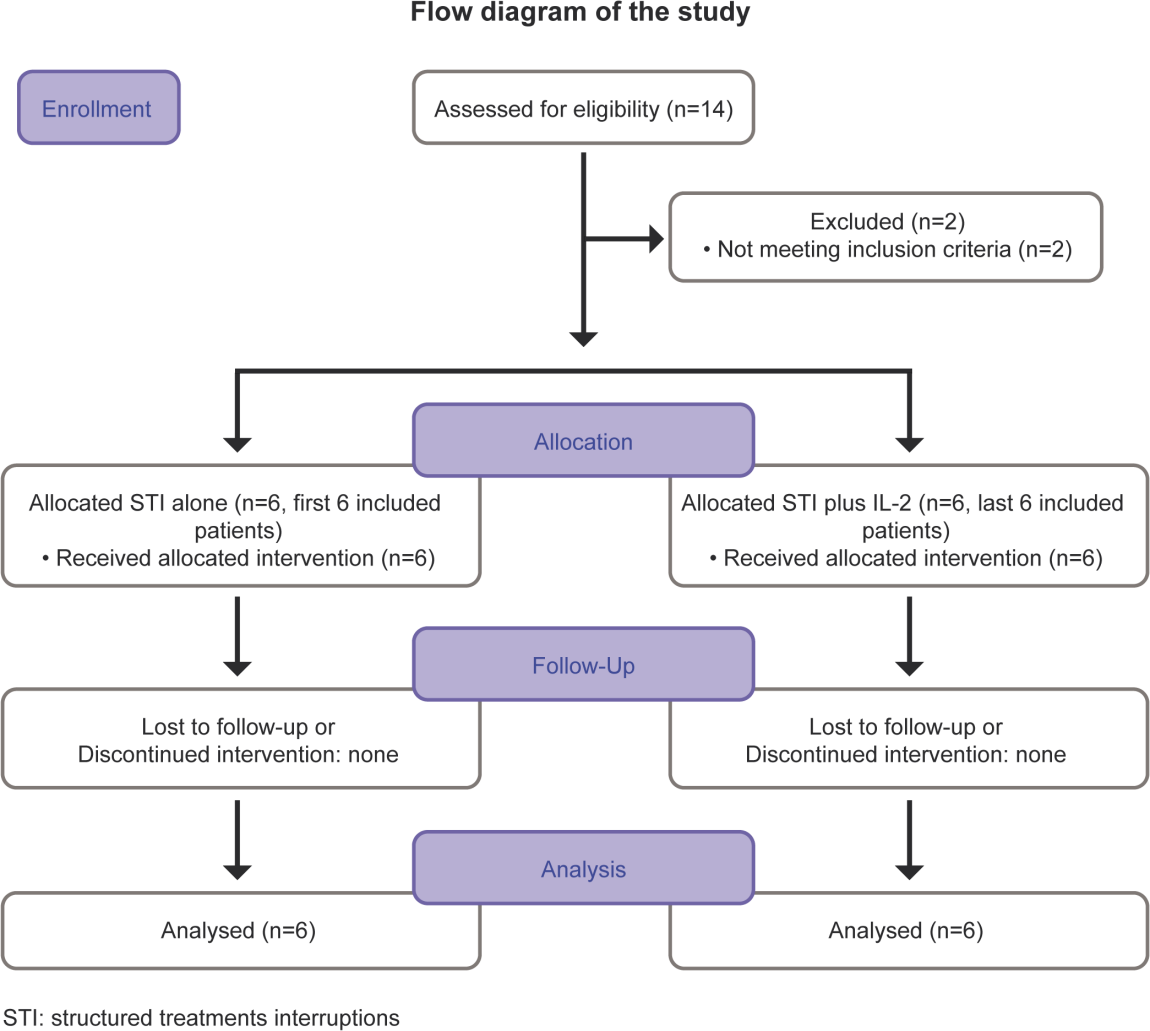
Flow diagram of the trial.

Enrolment started in March 2000 and finished in November 2001. Long-term follow-up for the last included patient finished in April 2012. All patients with PHI on stable effective ART for at least 12 months were invited to participate. The flow diagram of the study is shown in Fig 1.

Diagnosis of PHI was defined by:

-A detectable plasma viral load (PVL) or p24 antigen detection coupled with a negative or indeterminate LIA assay (according to CDC criteria), or
-A negative HIV-1 EIA in the preceding 90 days, or
-A positive EIA and LIA assay with acute retroviral syndrome in the 90 days preceding the start of ART plus documented negative HIV-1 EIA within the previous year.

Date of infection was assumed to have occurred two weeks before the onset of acute symptoms. In asymptomatic patients this date was calculated as the midpoint between the last negative and first positive test.

All patients started stavudine, lamivudine and non-boosted-indinavir at usual doses, according to recommendations at that time, within 90 days of HIV exposure, and had to show good virological and immunological responses, defined as undetectable PVL (<20 copies/mL in the last two controls), CD4 T cells higher than 500 cells/mm3 and a CD4/CD8 ratio >1 in the 8 months prior to enrolment.

### Study Design

**Fig 2:**
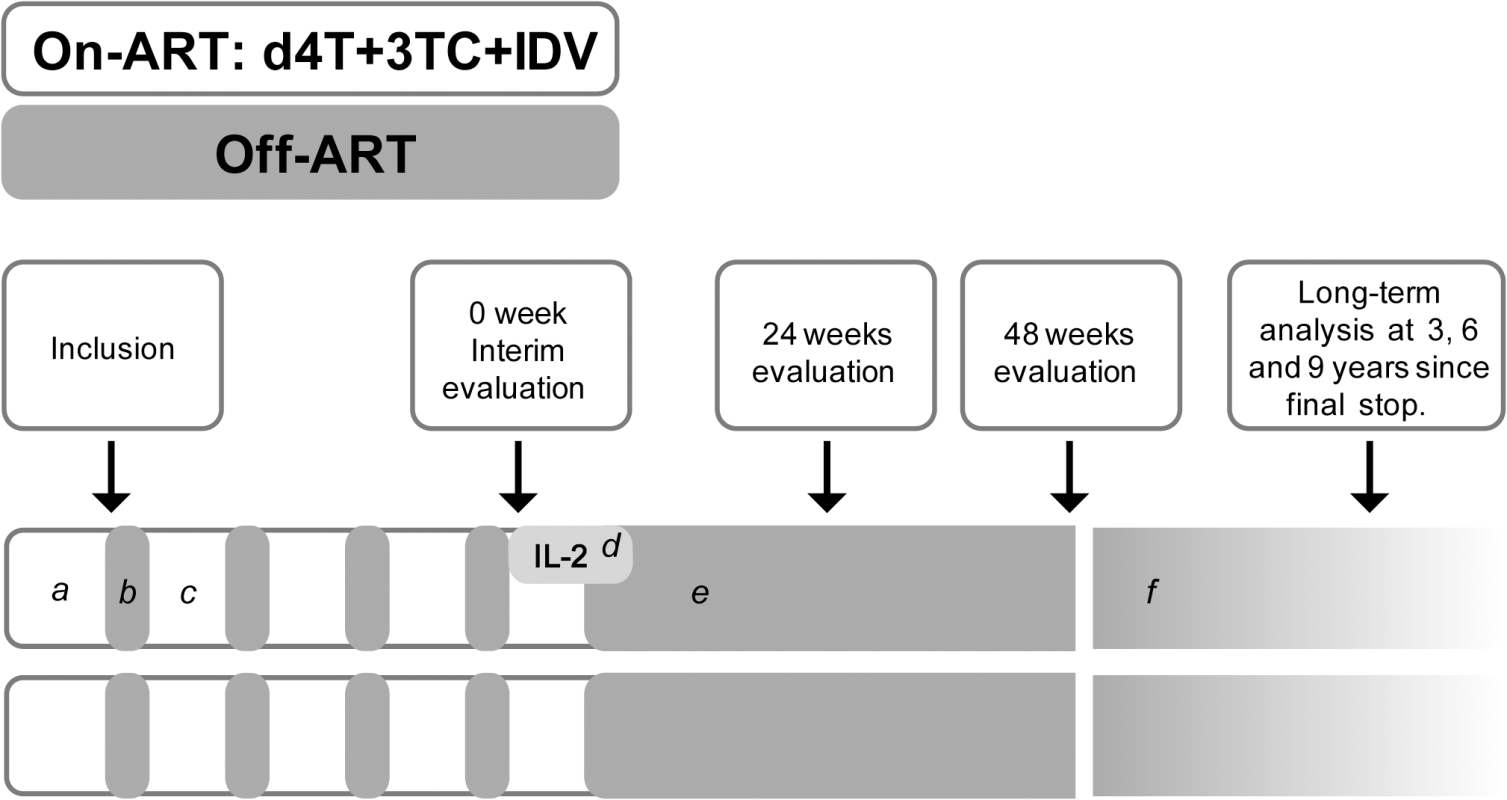
Design of the study. Footnote: (a) Eligible individuals were patients treated within 90 days of HIV exposure on stable ART for at least 12 months. Patients had to show good virological and immunological responses (at least 2 viral load <20 copies/mL and CD4 T cells higher than 500 cells/mm3 with a CD4/CD8 ratio >1). All individuals started four structured treatment interruption cycles (b) of 8 week each (*off-ART*), followed by four cycles (c) of treatment (*on-ART*). After the last treatment interruption (week 0) 6 patients started self-administrated IL-2 at a dose of 750,000 UI/m2 daily for 6 months (d). Treatment was interrupted in both arms when patients reached viral load <20 copies/mL (e). Analyses were performed at 24 and 48 weeks and after a long term follow-up period (9 years). During this period (f) treatment was restarted in patients whose CD4 cell count dropped below 350 cell/mm3.

The study design included two phases (Fig 2). The first phase consisted of four STIs of 8 weeks each (*off-ART*), separated by at least 16 weeks of treatment–or the time necessary to return to PVL <20 copies/mL—(*on-ART*). At the end of 4^th^ *off-ART* cycle (week 0), an interim evaluation was performed and the second phase initiated. During the second phase, the first 6 patients received ART until they reached PVL<20 copies/mL, discontinuing thereafter (final stop). The last 6 patients received ART and low doses of IL-2. ART was stopped after reaching PVL<20 copies/mL (final stop) and IL-2 after 6 months of treatment. IL-2 was prescribed at a dose of 750,000 UI/m^2^ daily and was self-administrated in all patients after training with a specialized nurse; administration was checked and verified during follow-up visits.

In both groups, ART was resumed in patients whose CD4 cell count dropped below 350 cells/mm3 in two consecutive determinations or in patients who developed opportunistic infections. Evaluations were performed at interim analysis (end of 4^th^ off-ART, week 0) and 24 and 48 weeks after the end of 4^th^ off-ART. A long-term follow-up analysis was also performed up to 9 years from the final stop. It included survival rate, clinical events, time to resume ART, CD4-CD8-CD4/CD8 ratio. This was performed, as standard of care, during follow-up visits.

### Measurements and evaluation

At enrolment the investigators recorded patient medical histories and performed a clinical examination. During the follow-up, clinical status and adverse events were recorded. Safety parameters, plasma HIV-1 RNA load, and CD4 and CD8 T cell counts were obtained at weekly intervals during off-ART and until PVL lowered below 20 copies/mL and monthly thereafter. Viral reservoir (HIV proviral DNA) was not performed.

### Laboratory methods

HIV serology was determined with a microparticulate enzyme immunoassay (MEIA) AxSYM system (Abbott Laboratories, North Chicago, IL) and confirmed with LIA (Inno-LIA HIV I/II Score. Innogenetics. Ghent, Belgium). PVL was determined using the Amplicor HIV-1 Monitor Ultra Sensitive Specimen Preparation Protocol Ultra Direct Assay (Roche Molecular Systems, Inc., Somerville, NJ) with a limit of detection of 20 copies/mL. Those samples below the detection limit were retested with a lower limit of detection of 5 HIV-1 RNA copies/mL as described[32]. HIV-1-RNA quantification in tonsillar tissue was performed in each patient at baseline as previously described[33]. Subpopulations of CD3+, CD4+, and CD8+ T cells, as well as proportion of CD8/CD38+ cells were determined by flow cytometry.

Genotypic mutations of both reverse transcriptase (RT) and protease (PR) genes from viral RNA were tested using the ViroSeq HIV Genotyping System v.2 (Abbott Laboratories, North Chicago, IL) and ABI3100 sequencer. Samples for genotype were drawn at baseline and when patients presented PVL higher than 1,000 copies/mL after the final ART discontinuation. Resistance mutations were re-interpreted according the HIV2007 IAS consensus. Viral subtype was inferred comparing the “fasta sequence” in REGA HIV-1 Subtyping Tool—Version 2.0[34]. The HLA class I genotype was determined by reverse sequence-specific oligonucleotide (SSO) (RELIDynal, Madrid, Spain). Allele definition was automatically assigned by the RELI SSO Pattern-matching Program software and was manually supervised. Tumoral necrosis factor (TNF) and soluble receptor for interleukin 2 (srIL2) levels were measured by EIA based techniques (Immunotech, France).

### Lymphocyte Proliferation Assays

Freshly isolated PBMCs were washed twice and resuspended at 2 × 10^6^/mL in a serum-free medium X-VIVO 10. Cultures were plated in triplicate at 2 × 10^5^/well in 7-day assays, in 96 round-bottomed microplates (TPP, Trasadingen, Switzerland). Cells were cultured in the absence or presence of Pokeweed mitogen 10 μg/mL (Sigma) and 5 μg/mL of HIV-1 recombinant proteins gp160 and p24 (Protein Sciences, Meriden, CT). Incorporation of tritium-labeled thymidine was assessed for the last 18 hours of culture (Betaplate LKB, Wallac, Sweden). Results were expressed as mean counts per minute (cpm). The stimulation index (SI) was calculated for each sample using the formula: SI = mean cpm for cells with stimulus/mean cpm for cells without stimulus. Positive antigen-specific responses were defined as >3,000 cpm and SI >3. For analytical purposes, results were expressed as ‘positive’ or ‘negative’ CD4 proliferative responses to HIV-1 P24 protein.

### HIV-1-Specific CD8+ T-Cell Responses

An ELISPOT assay (enzyme-linked immuno spot assay) was used to measure HIV epitope-specific CD8+ T-cell interferon-release from cryopreserved PBMC samples[6]. A mean of 16 (range: 3–27) different HLA class I–restricted synthetic peptides from gag, pol, env, and nef proteins were tested in each individual according to the HLA genotype. Results were expressed as total Spot Forming Cells (SFC)/10^6 PBMC and considered as positive with more than 500 SFC/determination.

### Statistical analysis

The primary endpoint was the proportion of patients who maintained a PVL <3,000 copies/mL and CD4 cells >500/μL at 48 weeks from the end of the 4th STI (allocation to ART alone or ART plus IL-2, week 0). An interim analysis was performed at week 0 and at week 24.

Virological and immunological parameters during follow-up and changes from baseline were summarized by median and interquartile range (IQR) values. For the purpose of analysis, undetectable PVLs (<5 and <20 copies/mL) were considered equivalent to 5 and 20 copies/mL, respectively. The PVL values underwent log10 transformation before analysis.

The χ^2^ test and the Fischer exact test were used, as appropriate, to compare categorical variables. Continuous variables were compared between subgroups using the Mann-Whitney test. For long-term analysis and time free of ARV after final stop, Kaplan-Meier curves and the log-rank test were used. All p were considered significant at p<0.05. For missing values related to endpoints, the most recent available determination for this variable was considered and specifically mentioned in the results section. All statistical analyses were performed with Stata and SPSS software.

### Ethics Statement

The study was approved by the Hospital Clínic Ethics Committee Board, and by the Spanish Regulatory Agency (‘Agencia Española del Medicamento’). All participants gave their written informed consent before enrolment. Registration of the trial in clinicalTrails.gov (NCT02300623) was retrospectively performed (registration was not compulsory at the time of the study conception).

## Results

### Patient selection

Fourteen patients were enrolled (Fig 1). Two patients were prematurely discontinued. Twelve patients completed the protocol and were included in the final analysis. All patients included in this analysis completed the four STIs and the follow-up period up to 8 years from the 48 weeks analysis. The STI periods started on 3/27/2000 for the first patient. The last patient completed the 48 weeks of follow-up on 4/20/2004.

### PHI characteristics

Of the 12 patients, 10 were male, median age was 35 years (IQR 28.5–2.54) and the main risk category was homosexual/bisexual intercourse. PHI was symptomatic in 9 of 12 patients, and the median interval between the estimated date of infection and ART initiation was 11 weeks (IQR 5.5–12), median VL was 103,500 copies/mL (IQR 23,379–207,000), median CD4 cell count 536.5 cells/mm3 (IRQ 391–612.5) and CD4/CD8 ratio 0.735 (IQR 0.425–0.970), all variables being comparable between the two groups. All patients were infected with HIV-1 B subtype virus, none had evidence of transmitted antiretroviral drug resistance and all patients were in Fiebig 4 phase at the time of ART initiation. None of the patients showed genetic variation in the CCR5 co-receptor gene, HBV or HCV co-infection.

### Characteristics at inclusion

The median time of treatment was 22.5 months (range 12–45), RNA-VIH-1 was below 5 copies/mL in 11/12 patients (patient #14 had 12 copies/mL), and the median CD4 count was 1,067 cells/mm^3^, (IQR 848–1328) with a median increase of 487 cells/mm^3^ since PHI. All but one patient had tonsillar RNA-HIV-1 below 40 copies/mg (patient #17 had 104 copies/mg). Baseline data at inclusion are shown in Table 1.

**Table 1:** Baseline characteristics at inclusion and CD4 T cells and viral load during STI.

		Total (n = 12)		STI (n = 6)		IL-2 (n = 6)		p value*
PHI characteristics		n	%	n	%	n	%	
Risk Category								0.545
	MSM/bisexual	8	67	5	83	3	50	
	Heterosexual	3	25	1	17	2	33	
	IDU	1	8			1	17	
Gender								1
	Male	10	83	5	83	5	83	
	Female	2	17	1	17	1	17	
		median	IQR	median	IQR	median	IQR	p value**
Age (years)		35	28.5; 42.5	36.5	28; 47	32.5	29; 36	0.52
								
CD4 T cells/mm^3^		1,067	848; 1328	1,064	566; 1415	1,067.5	1012; 1097	0.973
CD4/CD8 ratio		1.59	1.31; 1.80	1.64	1.58; 1.78	1.31	1.10; 1.86	0.648
CD4 T cells and viral load during STI								
CD4 T cells (Nadir during STI) cells/mm^3^								
1^st^ STI		641	606; 776	666	626; 804	637	550; 749	0.485
2^nd^ STI		546	443; 899	718	383; 870	597	114; 667	0.486
3^rd^ STI		681	448; 870	586	465; 1040	738	106; 823	0.699
4^th^ STI		592	347; 916	592	433; 968	528	204; 730	0.485
HIV RNA (Peak during STI) copies/mL								
1^st^ STI		150,500	25,650; 364,500	78,250	21,100; 596,000	363,500	30,200; 182,000	0.394
2^nd^ STI		23,250	5,260; 50,450	7,780	130; 26,000	42,000	10,400; 197,000	0.1
3^rd^ STI		19,200	1,655; 112,200	14,400	2,020; 19,200	98,350	1,290; 558,000	0.31
4^th^ STI		20,000	1,360; 54,650	13,740	1,240; 42,400	22,100	1,480; 197,000	0.485

*Fisher's exact test

**Wilcoxon Rank Sum test

### Primary endpoint

Only one out of 12 patients (in the STI group) maintained VL<3000 copies/mL and CD4 cells >500 cells/mm3 without ART at 48 weeks.

### Viral dynamics during the STI cycles and follow-up period

**Fig 3:**
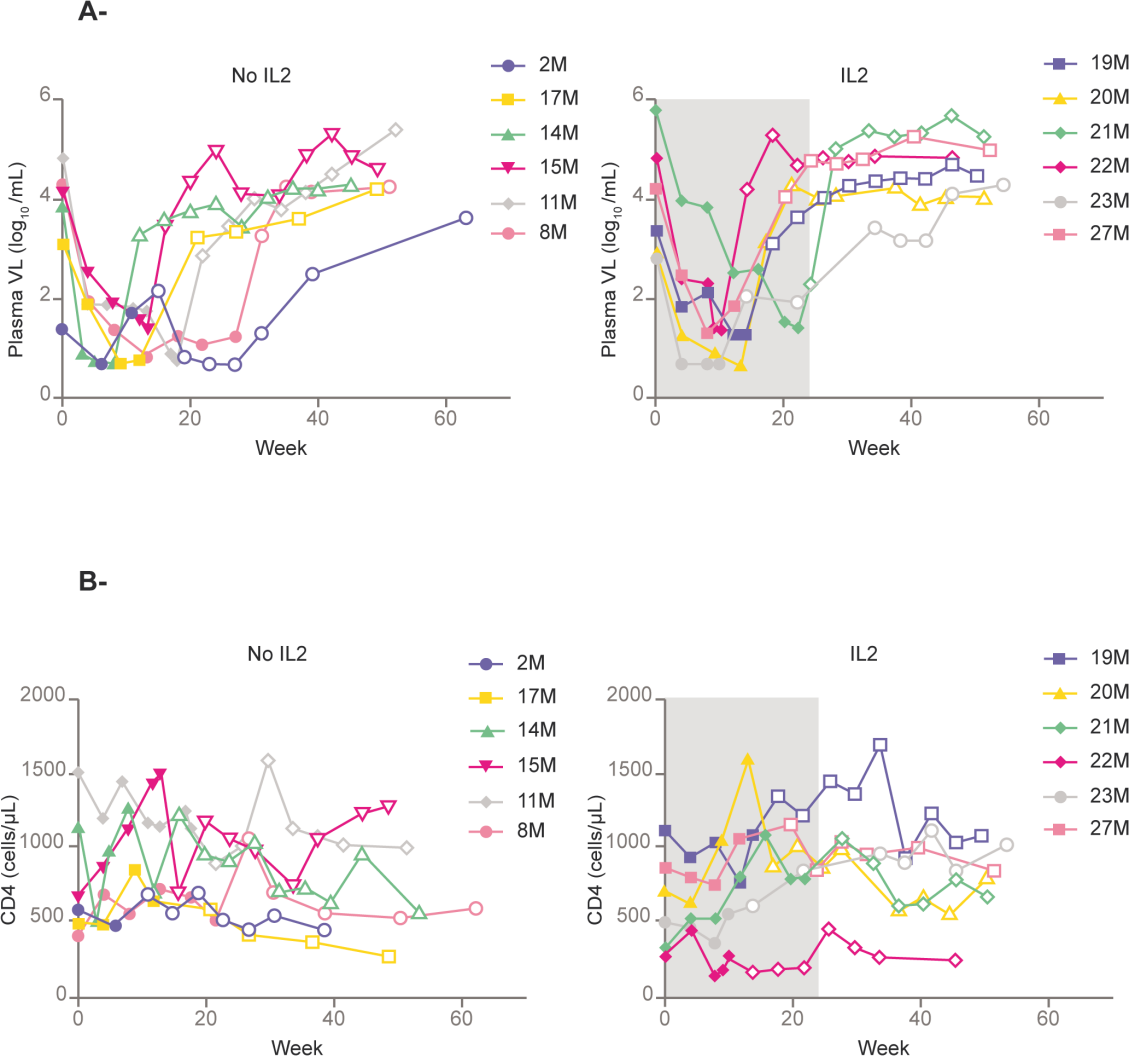
A) Plasma HIV Viral Load evolution after 4^th^ STI cycle. B) CD4+ T cell evolution after 4^th^ STI cycle. Footnote: The period of IL-2 administration is shown in grey. The filled points represent periods on ART. Empty points are determinations without ART.

Viral load rebounded during STI and at follow-up in all patients except for patient #2, who maintained viral load <5 copies/mL until 48 week after the ART interruption. The peak of PVL rebound was highest in the first STI, PVR rose to 150,500 copies/mL (IQR, 25,650–364,500 copies/mL) and decreased to stable levels during the following off-ART cycles: 23,250 (5,260–50,450) copies/mL in 2^nd^, 19,200 (1,655–112,200) copies/mL in 3^rd^ and 20,000 (1,360–54,650) in 4^th^ (Table 1). There was a trend to an increased doubling time of PVL during the off-ART cycles (11.07 days in the 1^st^ off-ART cycle and 24.45 days in the 4^th^ p = 0.09). After resuming ART, all patients showed rapid declines in PVL in each treatment phase. The number of patients fulfilling *Responder* criteria was 2 out of 12, 4 out of 12, 5 out of 12 and 4 out of 12 patients during 1^st^ to 4^th^ off-ART cycle respectively. After 24 weeks of follow-up, 2/6 patients in the IL-2 arm and 2/6 patients in the non-IL-2 arm maintained PVL <3,000 c/mL and after 48 weeks only one (in the STI group, although reported at 40 weeks) out of 12 patients did. Between weeks 40 and 60, all patients presented VL around log4. VL dynamics after 4^th^ STI are shown in Fig 3 and Table 2.

**Table 2:** Inflammation, immune activation, immunological and virological evolution following the end of 4^th^ STI (week 0) until week 48 of follow-up.

	Week 0 (end 4^th^ STI)		End of ART (final STOP)		Week 24		Week 48	
CD4 (cells/mm^3^)								
STI group	622	(514–1,167)	1,116	(669–1,297)	928	(601–1,079)	668	(514–1,033)
IL-2 group	613	(278–864)	933	(559–1092)	865	(832–906)	690	(669–936)
CD4/CD8 ratio								
STI group	1.05	(0.90–1.60)	1.55	(0.80–1.60)	1.35	(1.00–1.55)	1.05	(0.75–1.30)
IL-2 group	0.83	(0.77–1.00)	1.1	(0.92–1.87)	0.93	(0.67–1.28)	0.77	(0.48–0.87)
CD8/CD28+ (%)								
STI group	57	(37–60)	56	(39–65)	59	(33–61)	52	(34–62)
IL-2 group	49	(36–64)	38	(34–58)	47	(26–50)	51	(42–53)
CD8/CD38+ (%)								
STI group	57	(52–64)	47	(52–64)*	47	(52–64)	53	(44–63)
IL-2 group	46	(36–57)	61	(53–67)*	56	(53–69)	47	(44–59)
HIV Viral Load (log10/mL)								
STI group	4.13	(3.64–4.64)	<1.30		3.36	(2.89–3.93)	4.26	(4.15–4.60)
IL-2 group	3.98	(2.96–4.83)	<1.30		4.52	(3.76–5.08)	4.58	(4.19–5.01)
HIV Viral Load<3000 copies/mL								
STI group		2 (33%)	N/A		2(33%)		1(17%)	
IL-2 group		3 (50%)	N/A		2(33%)		0	
TNF levels (pg/mL)								
STI group	30	(29–33)	24	(21–26)*	26	(16–29)*	19	(17–32)
IL-2 group	36	(21–42)	39	(37–49)*	42	(27–73)*	44	(29–64)
srIL-2 (pM)								
STI group	61	(61–82)	59	(35–60)*	60	(39–67)*	76	(39–91)
IL-2 group	76	(42–104)	124	(116–249)*	124	(101–127)*	114	(101–132)
Specific CD4 responses (positive antigen-specific anti-P24 protein responses) number of patients/total patients								
STI group	2 out of 6				1 out of 6		none	
IL-2 group	1 out of 6				2 out of 6		1 out of 6	
Specific CD8 responses (>500 SFC/10E6 PBMC) number of patients/total patients								
STI group	4 out of 6*				1 out of 6		none	
IL-2 group	none*				none		none	

Values are median and IQR

*p<0.05

ART: Antiretroviral treatment

STI: structured treatment interruptions

srIL-2: seric receptor for IL-2

SFC: Spot Forming Cells

PBMC: Peripheral blood mononuclear cells

### Lymphocyte dynamics and cytokine levels during STI cycles and follow-up period

CD4 cells dropped in every STI but never below 500 T CD4 cells/μl. Immunological, virological and cytokine evolution at the end of 4^th^ STI (interim evaluation), the end of ART (Final Stop), week 24 and week 48 is shown in Table 2. Briefly, CD4 cell count, CD4/CD8 ratio and CD8-CD28+ cells were all comparable for both groups at the end of 4^th^ STI, the end of ART (final stop), week 24 and week 48. However, the proportion of CD8-CD38+ cells was significantly higher at the end of ART (final stop) for the IL-2 group. TNF and srIL-2 were also higher in the IL-2 group and the end of ART (final stop) and at 24 weeks of follow-up. These differences vanished at 48 weeks of follow-up. CD4 cell dynamics after 4th STI and until week 48 are shown in Fig 3 and Table 2.

### Specific CD4 and CD8 immune responses

None of the 12 patients presented specific CD4 responses before STI. This response increased to 5/6 patients at 3rd STI cycle for the STI group and to 4/6 patients at 2nd STI for the IL-2 group. However, they decreased progressively for both groups (2/6 for the STI group and 1/6 for the IL-2 group at week 0-end of 4th STI-), and, at week 48, all but one patient in the IL-2 group had lost these HIV-specific CD4 responses.

Regarding HIV-specific CD8 responses, 1/12 presented specific CD8 responses before STIs (in the IL-2 group), which also increased in both groups during STI. The number of patients presenting HIV-specific CD8 responses was comparable in both groups in 1st, 2nd and 3rd STI (p = 0.558, p = 1, p = 0.558 respectively). However, at week 0-end of 4th STI- these responses decreased to zero patients for the IL-2 group compared to 4 in the STI group (p = 0.014), and at 48 weeks of follow-up, they were absent in all 12 patients (Table 2).

Considering the 12 patients together, there were no statistically significant differences in the VL doubling time among those patients eliciting a strong CD8 response compared to those eliciting a weak CD8 response (p = 0.530, p = 0.180, p = 1, p = 0.283 for 1^st^, 2^nd^, 3^rd^, 4^th^ STI respectively).

### Development of ART resistance mutations

After the 4 STI cycles, only one patient developed K70R resistance mutation.

### Low IL-2 dose tolerability

Overall injections were well tolerated, but all patients presented mild injection-site reactions. One patient presented cellulitis. No adverse effects on liver tests, red blood cells, white blood cells or lipid profile were seen (data not shown).

### Long-term clinical and immunological follow-up

**Fig 4:**
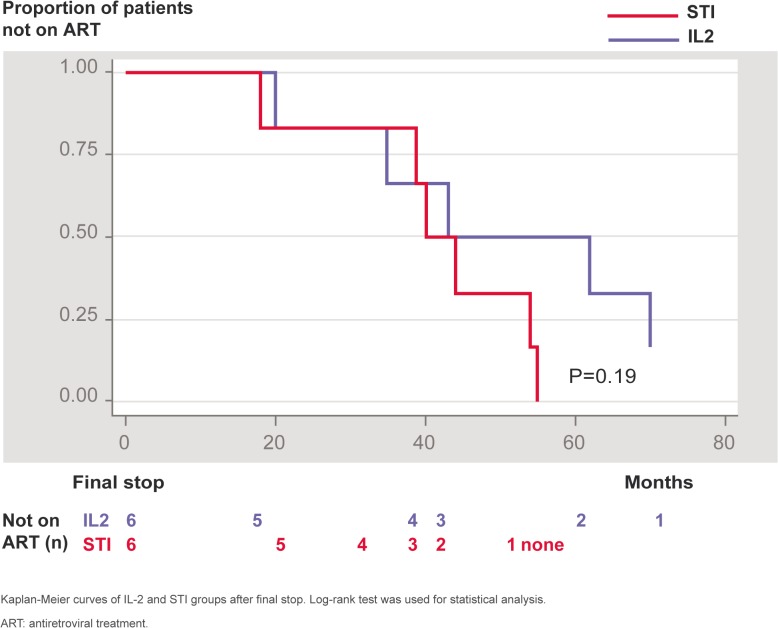
Proportion of patients not receiving ART after the final stop.

One patient in the IL-2 group died at 7 years and 10 months following the final stop due to metastatic colon cancer, aged 60. Overall 11/12 patients were alive at 9 years after the final stop. No other patient presented an AIDS-related opportunistic infection or neoplasm or other non-AIDS defining cancer. All patients in the STI group resumed ART at the end of the long-term follow-up and 5 out of 6 in the IL-2 group (one patient did not need to resume ART during the 9 years following the final stop, keeping CD4 cells consistently between 600 and 1100 cells/μl and VL to range around log4). Most of the patients in both groups resumed ART with a NNRTI (efavirenz)-based regimen. At the time of resuming ART, CD4 cells were comparable: 291/mm^3^ for the STI group and 325/mm^3^ for the IL-2 group (p = 0.855); CD4 percentage and CD4/CD8 ratios were also comparable for both groups. Immunological parameters remained comparable during long-term follow-up. Nine years after final stop the number of CD4 and CD8 cells and CD4/CD8 ratio were not different between groups: CD4 cells = 769.5/mm^3^ for the STI group and 844.5/mm^3^ for the IL-2 group (p = 1), CD4 percentage = 38.88 for the STI group and 38.7 for the IL-2 group (p = 0.394) and CD4/CD8 ratio = 1.20 for the STI group and 0.95 for the IL-2 group (p = 0.522). Time to resume ART after the final stop is shown in Fig 4. There was a trend to delayed resuming of ART in the IL-2 group. At 60 months after final stop, 3 out of 6 patients in the IL-2 group were not on ART compared to none in the STI group, but the difference did not reach statistical significance (p = 0.19).

## Discussion

The most recently published reports suggest that a favorable immunological outcome is obtained when ART is initiated very early in the course of primary HIV infection [25, 35, 36]. Moreover, in recent years it has been shown to have had a big impact in transmission for patients with PHI/recent HIV infection, adding epidemiological aspects to the arguments for ART during this phase[35, 37, 38]. ART in primary infection may also improve patients’ quality of life[39]. However, although all guidelines suggest treating symptomatic PHI patients, the indication is less clear for those who are asymptomatic. When ART is initiated, most guidelines recommend lifelong therapy due to the risk of viral rebound (and, probably, loss of any advantage) if treatment is stopped. In cases where ART is stopped, several immunological strategies have been evaluated as having impact on the clinical and immunological evolution of HIV infection and delaying the resuming of ART. Among these strategies, STI might boost HIV immunity, and several studies have analyzed this approach, including the use of STI alone or associated with other immune modulating therapies. A recently published study evaluated the impact of Peg-IFN and STI on PHI, but apart from lower peaks of HIV viremia during STI, no favorable immunological differences were seen in the long-term follow-up[31]. In our study, unfortunately, we also failed to prove any immunological benefit: at 48 weeks of follow-up all patients in both groups had lost the specific anti-HIV immune responses. According to our definition criteria of *Responder*, only one patient in the STI group had less than 3,000 copies of HIV-1 RNA, thus failing to show control of viral replication in both groups. We have seen no differences in the CD4 levels and viral load between groups. Thus, our 12 patients rebounded during the follow-up, with a relative decrease in the PVL with the following interruptions, but none of them evolved as a post-treatment controller, which has been calculated to occur in around 10% of the patients treated during PHI[25]. However, all our patients evolved clinically well when ART was resumed, thus there was not a deleterious long-term effect of STI. This is particularly important for the design of HIV functional-cure trials, in which, sooner or later, ART must be stopped to test spontaneous viral control but without harming patients not showing such viral control. However, we did not measured viral reservoirs, which could have been increased with STIs.

The IL-2 group showed higher immune activation and higher levels of inflammatory markers during the initial 24 weeks of follow-up. It is not clear whether this higher immune activation and inflammation impact the clinical outcome negatively in the long-term follow-up, but the only death, due to a non-AIDS defining cancer, was noted in the IL-2 group. Chronic inflammation and immune activation, among other factors, have been associated with non-AIDS defining cancers among HIV-infected patients[40]. However, in the studies conducted with IL-2 in chronic HIV-infection, with more than 3,000 patients, no increased incidence of cancer was seen[41]. On the other hand, there was a trend to delayed initiation of ART after the final stop in the IL-2 group but the numbers are too small to draw definite conclusions. The delayed resuming of ART did not prevent achieving equal immunological parameters (CD4, CD4 proportion and CD4/CD8 ratio) 9 years after the final stop between groups. However, in a life-long treatment scenario, this has very little clinical relevance. Indeed, nowadays most guidelines recommend an earlier initiation of ART, regardless of CD4 cell count.

It seems that the window to obtain an immunological advantage when treating PHI is very narrow, as suggested by a recent cohort study[36] in which patients reached 900 CD4 cells/μl more frequently when they started ART within 4 months of primary infection. In another recently published study, Goujard et al. suggest that the precocity of ART is not the only important factor: baseline immune response against HIV is a key factor to control HIV replication efficiently in a long-term follow-up after stopping ART[35]. In another study, however, apart from early ART during PHI, no other factor was found to be associated with long-term control of viremia[24]. The PRIMO-SHM trial investigated the impact of 24 or 60 weeks of ART compared to no-ART during PHI, and they concluded that a short cycle of ART might delay the indication for treatment later[42]. In the SPARTAC study, the conclusions were similar, but the time gained to resume ART was approximately the same for patients who were on treatment during PHI, thus the benefit was very limited[43]. Therefore, it seems clear that ART should be administered as early as possible to obtain the maximum immunological advantage, but whether the addition of another immunomodulating strategy (such as IL-2) may help ART in this setting remains unknown.

As far as we know, this study is the first to evaluate the effect of daily low-dose IL-2 in association with STI during PHI. It is also one of the few studies reporting such a long period of follow-up, giving a reliable view of the intervention effect. It has, however, several limitations. First, the small size of patients in both arms probably prevented any statistically significant difference being found in the time to resume ART. Secondly, the indication to resume ART was outside of the originally selected period of follow-up of the study, and it was driven by the clinical decision of the attending physician; however, CD4 cells at resuming were not statistically different among both groups, allowing us to compare them. Third, at the time of the design of this study, a VL lower than 3000 copies/mL and a CD4 cell count higher than 500 cells/mm3 was not considered an indication for ART. Nowadays most clinicians would treat these patients, since indications for ART have evolved and most guidelines recommend ART as of diagnosis, regardless of CD4 cell count or VL. Finally, due to the lack of clinical benefit in large clinical trials, the interest on IL-2 administration in HIV infection has significantly decreased.

In conclusion, in the short term, STI proved able to boost specific immune responses, but these responses were lost 48 weeks after stopping ART. IL-2 failed to boost the specific anti-HIV T-cell immune responses. There was an increased immune activation and inflammation in the IL-2 group that also vanished during follow-up. In the long-term follow-up, there was a trend to delayed need to resume ART in the IL-2 arm, not reaching statistically significant differences. Although considering that the potential delay of ART in a lifetime treatment might not be very relevant, this small study suggests that immunological interventions during PHI may impact the long-term outcome. Probably these results cannot be translated into clinical practice, but they may encourage further research in this topic. Larger studies are needed to prove whether IL-2 or other immune modulating strategies may be useful in the setting of PHI.

## Supporting Information

S1 CONSORT Checklist CONSORT 2010 check list. (DOC)

S1 Protocol Original Protocol. (DOC)

S2 Protocol Protocol, English Translation. (DOC)
