# Supplementary material for: Structured Treatment Interruptions and Low Doses of IL-2 in Patients with Primary HIV Infection. Inflammatory, Virological and Immunological Outcomes
Source: PLoS One. 2015 Jul 17;10(7):e0131651. doi: 10.1371/journal.pone.0131651 (PMC4506046; doi:10.1371/journal.pone.0131651)
Supplement: S1 Protocol — (DOC) [file pone.0131651.s002.doc]

*PROTOCOLO DE ENSAYO CLÍNICO CON MEDICAMENTOS:*

| ESTUDIO PILOTO PARA LA VALORACIÓN DE LA TERAPIA INTERMITENTE Y EL EFECTO DE LA INTERLEUCINA-2 EN PACIENTES VIH POSITIVOS TRATADOS DURANTE LA PRIMOINFECCION |
| --- |

Protocolo: PHI-IL2

Versión: 1.0 (13/09/2001)

Servicio de Infecciones

Hospital Clínic.

Villarroel, 170. 08036 Barcelona

Tel. 93 2275430

Fax: 93 4514438

**1. Resumen.**

1.0. Tipo de solicitud:

Ensayo clínico en una nueva indicación.

1.1. Identificación de los promotores:

| Dr. José Mª Miró Meda  Servicio Enfermedades Infecciosas  Hospital Clínic  Villarroel, 170  08036 Barcelona | Dr. José Mª Gatell Artigas  Servicio Enfermedades Infecciosas  Hospital Clínic  Villarroel, 170  Barcelona |
| --- | --- |

1.2. Título:

Estudio piloto para la valoración de la terapia intermitente y el efecto de la interleucina-2 en pacientes VIH positivos tratados durante la primoinfección

1.3. Código del protocolo: PHI-IL2

1.4. Investigadores principales:

| Dr. José Mª Miró Meda  Servicio Enfermedades Infecciosas  Hospital Clínic  Villarroel, 170  Barcelona | Dr. José Mª Gatell Artigas  Servicio Enfermedades Infecciosas  Hospital Clínic  Villarroel, 170  08036 Barcelona |
| --- | --- |

1.5. Centros en los que se prevé realizar el ensayo:

Instituto de Enfermedades Infecciosas e Inmunología Clínica

Hospital Clínic

Villarroel, 170

08036 Barcelona

1.6. Comités Éticos de Investigación Clínica que han aprobado el ensayo:

El ensayo ha sido presentado para su aprobación por el CEIC de la Corporació Sanitària Clínic.

1. Nombre del responsable de monitorización:

Dra. Anna Cruceta Arbolés

Servicio de Infecciones

Hospital Clínic. Barcelona.

1.8. Fármaco experimental:

Aldesleukina (Interleucina 2 recombinante) (IL-2) Proleukin®, Chiron

Viales de 18 MUI

Grupo terapéutico L01F1A

1.9. Fase del ensayo: Fase III.

1.10. Objetivo principal:

Valorar la respuesta virológica, la respuesta citotóxica específica frente a VIH y el punto de estabilización de la viremia tras el 4º ciclo de interrupción del tratamiento antirretroviral (TARV), y la proporción de pacientes que no requieren TARV al final de estudio tras la administración de dosis bajas de IL-2 por vía subcutánea.

1.11. Diseño experimental:

Estudio clínico piloto, prospectivo, unicéntrico, abierto, no comparativo

1.12. Enfermedad en estudio:

Infección por el VIH-1.

1.13. Variables principales de valoración:

Proporción de pacientes que presentan carga viral (CV) plasmática superior a 3 veces el valor basal (previo TARV), o bien CV >20 copias/mL si la CV basal era < 10 copias/mL.

Proporción de pacientes con buena respuesta citotóxica tras el 4º ciclo comparado con el inicio del tratamiento

Poporción de pacientes que tras la administración de IL-2 no equieren TARV tras finalizar el estudio.(> 350 celulas CD4 o con CV<55.000 copias/mL)

1.14. Población en estudio:

Se incluirán un total de 8 pacientes ambulatorios, seropositivos para HIV que hubieran comenzado tratamiento antirretroviral de alta actividad (HAART) durante la primoinfección.

1. Duración del tratamiento:

El tratamiento se administrará a partir de la primera interrupción de tratamiento ARV diariamente a dosis de 125.000 MUI/m2 por vía subcutánea durante 6 semanas. Después de completar 4 ciclos de 6 semanas, el tratamiento con IL-2 se suspenderá. El seguimiento de los pacientes será de dos años.

1.16. Calendario y fecha prevista de finalización:

Se prevé iniciar el estudio, una vez obtenidos los permisos, en noviembre de 2001. El periodo de reclutamiento será de 2 meses, y el seguimiento se prolongará durante dos años. Se prevé que el estudio finalice en diciembre de 2003.

***

**2. Indice.** pág.

1. Resumen 2

2. Indice 5

3. Información general 6

4. Justificación y objetivos 8

5. Tipo de ensayo y diseño 12

6. Selección de los sujetos 13

7. Descripción del tratamiento 15

8. Desarrollo del ensayo y evaluación de la respuesta 17

9. Acontecimientos adversos 19

10. Aspectos éticos 21

11. Consideraciones prácticas 22

12. Análisis estadístico 23

13. Bibliografía 25

Anexos:

Anexo I. Cuaderno de recogida de datos (CRD).

Anexo II. Hoja de información para el paciente.

Anexo III. Hoja de consentimiento por escrito.

Anexo IV. Declaración de Helsinki.

Anexo V. Hoja de notificación de acontecimientos adversos.

Anexo VI. Seguro de responsabilidad civil.

Anexo VII. Información de los productos en estudio.

**3. Información general.**

A. Identificación del ensayo.

1º Código: PHI-IL2

2º Título: ESTUDIO PARA LA VALORACIÓN DE LA TERAPIA INTERMITENTE EN PACIENTES VIH POSITIVOS TRATADOS DURANTE LA PRIMOINFECCION

B. Tipo de ensayo clínico:

Ensayo clínico en una nueva indicación.

C. Descripción de los productos en estudio:

Denominación genérica, nombre comercial, forma farmacéutica y composición:

Aldesleukina (Interleucina 2 recombinante) (IL-2) Proleukin®, Chiron

Viales de 18 MUI

D. Datos relativos a los promotores.

| Dr. José Mª Gatell Artigas  Servicio de Infecciones  Hospital Clínic  Villarroel, 170  08036 Barcelona | Dr. José Mª Miró Meda  Servicio Enfermedades Infecciosas  Hospital Clínic  Villarroel, 170  Barcelona |
| --- | --- |

Teléfono: 93 227 54 00 Fax: 93 451 44 38

E. Director técnico responsable de la elaboración de las muestras:

El del laboratorio fabricante (Chiron).

F. Identificación del monitor:

Dra. Anna Cruceta Arbolés

Servicio de Infecciones

Hospital Clínic. Barcelona.

G. Investigadores principales:

| Dr. José Mª Miró Meda  Servicio Enfermedades Infecciosas  Hospital Clínic  Villarroel, 170  Barcelona | Dr. José Mª Gatell Artigas  Servicio de Infecciones  Hospital Clínic  Villarroel, 170  08036 Barcelona |
| --- | --- |

H. Centros y Comités Éticos que han informado favorablemente:

El ensayo ha sido presentado para su aprobación por el CEIC de la Corporació Sanitària Clínic.

1. Duración prevista del ensayo:

Se prevé iniciar el estudio, una vez obtenidos los permisos, en noviembre de 2001. El periodo de reclutamiento será de 2 meses, y el seguimiento se prolongará durante dos años. Se prevé que el estudio finalice en diciembre de 2003.

***

**4. Justificación y objetivos.**

1. *Inmunidad especifica frente al VIH y terapia intermitente.*

La terapia antirretroviral altamente activa (Highly Active Antiretroviral Therapy = HAART) ha reducido la incidencia de las enfermedades definitorias de SIDA en aproximadamente un 85% [1]. Sin embargo esto supone tratamiento con al menos tres fármacos de forma indefinida, ello implica incomodidad, padecimiento de efectos secundarios y un alto coste económico. Hasta el momento las tentativas de parar el tratamiento antirretroviral (TARV) han fracasado. Incluso en pacientes en los que la CV ha permanecido indetectable durante meses, la retirada del tratamiento ha sido invariablemente seguida del rebote de la CV en pocas semanas [2,3]. El rebote de la CV probablemente se inicia desde reservorios mantenidos en linfocitos infectados de forma latente [4].

Aunque en la mayoría de los pacientes el VIH destruye el sistema inmune, los escasos pacientes no progresores (Long Term Non Progressors= LTNP) toleran la infección durante años con aparentemente pocos efectos. En estos pacientes los CD4 permanecen en el rango de la normalidad y la CV es muy baja o indetectable. La mayoría de LTNP tiene una buena y persistente respuesta linfocitaria citotóxica frente al VIH [5-8]. Sin embargo esta repuesta es transitoria en los pacientes que no son LTNP, permitiendo la proliferación continuada del VIH que finalmente resulta en la inmunosupresión. [9]

¿Es posible inducir CTLs en pacientes que de otro modo progresarían hacía la inmunodeficiencia?, ¿Controlarían estos pacientes la proliferación viral después de retirar el tratamiento?. Hay datos preliminares que indican que puede ser posible:

-Los pacientes tratados muy pronto en el curso de la infección (durante la primoinfección o poco después) desarrollan una poderosa y duradera respuesta citotóxica contra el VIH [10].

-Se conoce el caso de 2 pacientes con mala adherencia a la terapia que pararon el tratamiento, lo que causó un rebote en la CV, posteriormente reintrodujeron el tratamiento por un periodo variable y finalmente suspendieron la terapia indefinidamente. En ambos la CV permaneció indetectable durante 14 y 21 meses sin ningún TARV. En lo dos casos ambos presentaron una buena y amplia respuesta citotóxica frente al VIH que probablemente fue reforzada por el rebote de la CV durante la primera interrupción del tratamiento. Se valoró la respuesta citotóxica cuando los paciente estaban sin tratamiento y esta había permanecido alta [11].

-Otro paciente tratado con ddI , hidroxiurea e indinavir paró el tratamiento y presento un rebote de la viremia. Se volvió a tratar con lo que la CV bajó y se volvió a parar el tratamiento. La viremia del paciente no rebotó y permanece así después de mas de un año sin tratamiento. El paciente presentó una buena respuesta citotóxica frente al VIH [12]

-Tres pacientes con viremia estable fueron tratados con un una combinación que contenía hidroxiurea siguiendo el siguiente esquema: 3 semanas de tratamiento, 1 semana de interrupción seguido por 2 ciclos de 3 meses de terapia seguido de interrupción y reintroducción del tratamiento tan pronto como la CV rebrotara (>5000 copias/mL). La CV se hizo indetectable (<400 copias/mL) después de cada reintroducción del tratamiento. Los intervalos libres de recaída aumentaron de 7 días después de la primera interrupción hasta 37 días después de la 3º interrupción. [12].

-*Rhesus macaques* infectados con SIV/Mac251 (viremia basal 200000 copias/mL, 500000 copias/mL y 1,1 millón copias/mL) fueron tratados con hidroxiurea, DDI y un derivado del adefovir siguiendo un esquema intermitente (dos ciclos de 3 semanas de tratamiento seguido de la interrupción del tratamiento). Se permitió el rebote de SIV hasta >5000 copias/mL. Aunque la CV rebrotó, se controló en los tres casos y se estabilizó en un nivel bajo de viremia en ausencia de tratamiento. Este nivel fue >5000 copias/mL mantenido durante 6 semanas en la 1ª interrupción y bajó a <200 copias/mL mantenido durante 10 semanas como mínimo en la 3ª interrupción. Dos de los monos continúan sin recaída después de 4 y 6 meses respectivamente [12].

La interrupción intermitente del tratamiento se asocia con un refuerzo de la respuesta citotóxica frente al VIH que puede contribuir a prolongar la supresión de la CV cuando el tratamiento es retirado.

1. *Suspender el tratamiento de forma aguda es aceptablemente seguro*

La exposición crónica a niveles subterapéuticos de antiretrovirales puede seleccionar mutantes resistentes que llevan a una pérdida de eficacia. Sin embargo la interrupción brusca del tratamiento no parece estar asociado con la formación de resistencias. En los casos de interrupción del tratamiento la proliferación viral se origina a partir de los reservorios del virus que permanecen en los linfocitos T no activados. Numerosos estudios han demostrado que el virus que permanece en los reservorios es sensible al TARV [13].

Neumann et al. y Garcia et al. [2,3,3] han presentado estudios de interrupción de la triple terapia, con seguimiento de los pacientes hasta que la CV rebrotó y alcanzó niveles estables, y posterior reintroducción del TARV. En los 23 pacientes presentados el control de la CV fue como mínimo tan rápido durante el 2º curso de tratamiento que como en el 1º. Todos los pacientes tenían una CV <200 copias/mL a las 6 semanas del 2º curso de tratamiento.

1. *Efectos de la administración de IL-2*

Existen numerosos estudios que han combinado el TARV con tres o más fármacos antirretrovirales con ciclos de IL-2 administrados durante cinco días a dosis elevadas (18 millones de UI/día) o intermedias (3-6 millones de UI/día) por vía i.v. o vía s.c. cada 8 semanas (14-23). Este enfoque se ha utilizado en pacientes avanzados (con recuentos de linfocitos CD4+ < 200-500 células/µL) con el fin de incrementar la cifra de linfocitos CD4+, de mejorar otros parámetros inmunológicos y de reducir la apoptosis de los linfocitos T. Además se ha observado que la administración concomitante de dosis elevadas de IL-2 y TARV reduce el *pool* de linfocitos CD4+ latentes que contienen el DNA del VIH-1 integrado (21). Sin embargo, cuando se detiene el tratamiento antirretroviral se observa un rápido rebote de la carga viral del VIH-1 en plasma (22). Por otra parte, la IL-2 a dosis elevadas o intermedias tiene numerosos efectos secundarios, sobre todo cuando se administra por vía i.v. (24). Esta toxicidad es dosis-dependiente y no existiendo efectos adversos cuando se administra a dosis bajas. En este sentido, los efectos adversos fueron excepcionales en un estudio en pacientes con neoplasias relacionadas con la infección por el VIH-1 que recibieron dosis bajas de IL-2 (0.5-1.2 millones de UI/día) durante 3 meses y, por otra parte, no se observaron infecciones oportunistas ni incremento de la CV del VIH-1 en plasma, objetivándose una mejoría inmunológica de la respuesta Th1 (24).

El objetivo de administrar dosis muy bajas de IL-2 en los pacientes con una primoinfección por el VIH-1 tratados desde el inicio de la infección aguda se basa en un estudio del grupo del Dr. KA Smith. Es dicho estudio se administró durante 6 meses dosis muy bajas de IL-2 por vía s.c. en pacientes asintomáticos con una infección crónica por el VIH-1 que recibían tratamiento antirretroviral en monoterapia y tenían una CV del VIH-1 en plasma relativamente elevada. Los pacientes no tuvieron efectos secundarios, fueron capaces de auto-administrarse la IL-2 y de mantener su actividad cotidiana (31). Además se objetivó que con esta dosis de IL-2 se conseguían una serie de beneficios inmunológicos como son: 1) Una mejoría cualitativa del sistema inmune, medida por los test cutáneos de hipersensibilidad retardada a antígenos memoria; 2) Un aumento de linfocitos CD4+; 3) Una ausencia de cambios significativos de la CV del VIH-1 en plasma; y 4) Una ausencia de un incremento valorable de citocinas pro-inflamatorias en plasma (TNF-α, IFN-γ, GM-CSF) (31). La dosis terapéutica máxima sin toxicidad durante 6 meses se consiguió con la administración diaría por vía s.c. de 187,000-250,000 unidades/m^2^/día. Los niveles plasmáticos pico de IL-2 fueron de 22 pM que es suficiente para saturar ≥ 70% de los receptores de alta afinidad de IL-2 en las células T , B y NK, mientras que solo se uniría a un 2% de los receptores de baja afinidad de la IL-2. Este estudio también demostró que fue efectiva la administración de una dosis de 125,000 unidades/m^2^/día (25). En consecuencia, esta dosis muy baja y sin efectos secundarios de IL-2 parece ideal como inmunoterapia adicional al TARV para actuar selectivamente expandiendo *in vivo* los receptores de alta afinidad de la IL-2 en los linfocitos CD4+ y CD8+ una vez activados con un estímulo antigénico específico, en este caso el propio VIH-1 durante el periodo de la interrupción del TARV. Ello es posible dado que son los linfocitos CD4+ los que tienen receptores de las células T capaces de reconocer los antígenos que le presentan las células presentadoras de antígenos. En base a todos estos datos creemos que la combinación de IL-2 y las interrupciones estructuradas del tratamiento antirretroviral puede hacer viable y expandir la respuesta VIH específica de los linfocitos CD4+ y CD8+ una vez activados durante el rebote de la CV y permitir un control de la replicación viral del VIH-1 una vez suspendido el TARV después del cuarto ciclo. Esta hipótesis se ha comprobado recientemente en un modelo experimental en primates superiores en los que la administración de IL-2-Ig, que es funcionalmente mucho más eficiente que la IL-2 natural, aumentó de forma importante la respuesta inmune celular y humoral en los animales vacunados con DNA del VIH-1 y del SIV (26).

Objetivo general:

Valorar la respuesta virológica, la respuesta citotóxica específica frente a VIH y el punto de estabilización de la viremia tras el 4º ciclo de interrupción del tratamiento antirretroviral (TARV), y la proporción de pacientes que no requieren TARV al final de estudio tras la administración de dosis bajas de IL-2 por vía subcutánea.

Objetivos específicos:

- Determinar la proporción de pacientes con CV con un incremento de al menos 3 veces el valor basal (previo al TARV) (corresponde aproximadamente a un aumento de 0,5 log).
- Determinar la proporción de pacientes con respuesta citotóxica específica frente a VIH
- Comparar la cifra de estabilización de la viremia al parar el TARV comparando el final del 4º ciclo y el final del estudio con la viremia antes del inicio del HAART
- Determinar la proporción de pacientes que no requieren tratamiento antirretroviral al final del estudio.

***

**5. Tipo de ensayo y diseño.**

1. Fase de desarrollo: fase III.
2. Descripción del proceso de aleatorización:

No procede por ser un estudio no comparativo

c) Tipo de control y diseño:

Estudio clínico piloto, prospectivo, unicéntrico, abierto, no comparativo

d) Técnicas de enmascaramiento:

No procede; se trata de un ensayo abierto.

e) Periodo de seguimiento:

2 años

***

**6. Selección de los sujetos.**

a) Criterios de inclusión:

Se incluirán pacientes de ambos sexos con:

1. Diagnóstico de infección por VIH-1.
2. Edad igual o superior a 18 años.
3. Tratados con tratamiento antirretroviral HAART (compuesto por dos análogos de nucleósidos inhibidores de la transcriptasa inversa más un inhibidor de la proteasa) desde el momento de la primoinfección.
4. Carga viral <50 copias/mL (Roche Monitor Assay) en plasma por un tiempo mínimo de 8 meses.
5. Recuento de linfocitos CD4 siempre superior a 500 céls/μL (antes de parar el tratamiento).
6. Que, adecuadamente informados, otorguen su consentimiento por escrito para participar en el estudio y someterse a las pruebas y exploraciones que ello comporta.

b) Criterios de exclusión:

1. Pacientes que hayan respondido adecuadamente al tratamiento antirretoviral inicial.
2. Pacientes que hayan tomado en algún momento nevirapina o efavirenz.
3. Mujeres embarazadas, en periodo de lactancia, o aquellas que pretendan quedar embarazadas durante el periodo del estudio.

c) Número de sujetos previstos:

Se incluirán 8 pacientes ambulatorios

d) Método del cálculo del tamaño de la muestra y datos empleados:

Se trata de un estudio piloto, pues se desconocen datos de respuesta al tratamiento en este subtipo de pacientes con primoinfección por HIV. Los resultados de este estudio permitirán calcular la muestra necesaria para comparar plantear una comparación con poder estadístico adecuado.

e) Criterios de retirada y análisis previsto de las retiradas y abandonos:

Los pacientes podrán abandonar el estudio en cualquier momento sin tener que dar explicaciones. Se procurará, sin embargo, conocer la causa de los abandonos y anotarlo en el cuaderno de recogida de datos (CRD).

El investigador podrá también retirar a un paciente del estudio por ineficacia, acontecimientos adversos o cuando lo considere oportuno en beneficio del paciente. El motivo de la retirada se anotará en el CRD.

Todos los pacientes que, una vez asignados a un grupo de tratamiento, abandonen el tratamiento en estudio o sean retirados por cualquier causa, se incluirán en un análisis "por intención de tratamiento".

Los pacientes que no continúen acudiendo a la visita (pérdidas de seguimiento) serán considerados para el análisis hasta el momento en el que se produzca la pérdida.

f) Tratamiento de las pérdidas prerrandomización:

Los pacientes que cumplan los criterios de selección pero que, por cualquier motivo, no lleguen a ser incluidos en uno de los grupos de tratamiento previstos, serán registrados adecuadamente (datos personales, demográficos, etc.) pero no se incluirán en el análisis.

g) Duración aproximada del periodo de reclutamiento:

Se prevé que será necesario un periodo de 2 meses para reclutar 8 pacientes de las características descritas.

***

**7. Descripción del tratamiento.**

Los pacientes suspenderán el tratamiento antirretroviral (TARV) durante 8 semanas , y se volverá a introducir durante 8-12 semanas más pero añadiendo interleucina-2 durante 6 semanas.

El mismo proceso se repetirá hasta completar 4 ciclos. Al finalizar el cuarto ciclo los tratamientos con IL-2 y TARV se suspenderán si no se demuestra replicación viral hasta que la carga viral (CV) alcance el punto de estabilización. (Definición de punto estabilización: CV iguales en 3 determinaciones seguidas y separadas por mínimo 3 semanas)

a) Dosis, intervalo, vía y forma de administración y duración.

Interleucina-2 (IL-2), 125.000 unidades/m^2^/día, por vía subcutánea durante 6 semanas.

b) Criterios de modificación de pautas a lo largo del ensayo:

• INTOLERANCIA/TOXICIDAD

Interleucina-2: se evaluará en cada visita la tolerabilidad de la medicación para proceder a su suspensión en caso que la gravedad o intensidad de los efectos adversos así lo aconseje.

• MEDIDAS DE SEGURIDAD

Por la preocupación ante la eventual aparición de resistencias y pérdida de efectividad del tratamiento antirretroviral, se han diseñado varias medidas para asegurar que la terapia intermitente será inmediatamente retirada en caso de fallo:

-Análisis de la respuesta al tratamiento en las semanas antes de cada parada**.** La CV se medirá a las 7 semanas de re-iniciar el tratamiento en cada ciclo. El estudio se parará si de entre los pacientes buenos cumplidores más de 2 no alcanzan CV<50 copias/mL.

-La interrupción del tratamiento a la semana 64-80 se realizará solo en aquellos pacientes que muestren disminución de la CV al comparar la tercera interrupción con la primera.

-Se iniciará el TARV en los pacientes con una cifra de linfocitos CD4+ <350 células/µL o con una CV de VIH-1 en plasma >55.000 copias/mL en dos determinaciones consecutivas (27). En los pacientes asintomáticos con una cifra de linfocitos CD4+ entre 350 y 500 células/µL y/o una CV entre las cifras previas se valorará de forma individual si debe reiniciarse el TARV.

-Se contará con un comité de seguridad.

c) Tratamientos concomitantes:

Cualquier medicación concomitante deberá quedar reflejada en el CRD adecuadamente (detallando el producto, dosis, vía, día de administración, motivo del tratamiento, etc.).

1. Medicación de rescate:

Se iniciará el TARV en los pacientes con una cifra de linfocitos CD4+ <350 células/µL o con una CV de VIH-1 en plasma >55.000 copias/mL en dos determinaciones consecutivas.

La medicación de rescate deberá quedar reflejada en el CRD adecuadamente durante todo el periodo de seguimiento del estudio.

1. Normas de manejo de los fármacos en estudio:

Las especialidades farmacéuticas utilizadas en el estudio no requieren condiciones especiales de conservación o manejo, en condiciones normales de humedad y temperatura.

***

**8. Desarrollo del ensayo y evaluación de la respuesta.**

a) Variables principales de evaluación (actividad y eficacia):

- Determinar la proporción de pacientes con CV con un incremento de al menos 3 veces el valor basal (previo al TARV) (corresponde aproximadamente a un aumento de 0,5 log).
- Determinar la proporción de pacientes con respuesta citotóxica específica frente a VIH
- Comparar la cifra de estabilización de la viremia al parar el TARV comparando el final del 4º ciclo y el final del estudio con la viremia antes del inicio del HAART
- Determinar la proporción de pacientes que no requieren tratamiento antirretroviral al final del estudio.

b) Variables secundarias (seguridad y tolerabilidad):

- Descripción de acontecimientos adversos

c) Número y tiempo de las visitas y pruebas o exploraciones.

Las visitas médicas se realizarán a la semana del comienzo del estudio, posteriormente cada 15 días durante 4 meses y después cada 2 meses hasta el final del estudio. Durante los periodos de parada del tratamiento se le realizarán análisis semanales. Durante los periodos de tratamiento los análisis serán mensuales.

Se efectuará una biopsia amigdalar tanto al inicio como al final del estudio.

Ver calendario (pág. 18).

d) Duración del estudio:

Dos años y medio desde la inclusión del primer paciente. El período de reclutamiento debería ser de 2 meses y la duración del estudio de 24 meses después de la inclusión del último paciente.

Calendario de las visitas y pruebas o exploraciones.

| Determinación | 0 | 8 | 16[[1]](#footnote-2) | 24 | 32 | 40 | 48 | 56 | 64 | 72 | 80 | 88 | 96 | 104 | 112 | 120 | 128 | 136 | >136 |
| --- | --- | --- | --- | --- | --- | --- | --- | --- | --- | --- | --- | --- | --- | --- | --- | --- | --- | --- | --- |
| CV (límite de detectabilidad de 10) | a | a | a | a | a | a | a | a | a | a | a | a | a | a | a | a | a | a | Cada 4 semanas |
| Hemograma | a |  |  |  |  |  |  |  | a |  |  |  |  |  | a |  |  | a | Cada 8 semanas |
| CD4 | a |  |  |  |  |  |  |  | a |  |  |  |  |  | a |  |  | a | Cada 8 semanas |
| Estudio de histocompatibilidad [[2]](#footnote-3) | a |  |  |  |  |  |  |  |  |  |  |  |  |  |  |  |  |  |  |
| Función citotóxica frente al VIH y función CD4 helper[[3]](#footnote-4) | a |  | a |  | a |  | a |  | a |  |  |  |  |  | a |  |  |  | Cada 16 semanas |
| VIH-DNA integrado  En células mononucleares perifericas (CMP) | a |  | a |  | a |  | a |  | a |  |  |  |  |  | a |  |  |  | Cada 16 semanas |
| Conservación de plasma y CMP | a |  | a |  | a |  | a |  | a |  |  |  |  |  | a |  |  | a | Cada 8 semanas |
| Tejido linfoide^4^ | a |  |  |  |  |  |  |  |  |  |  |  |  |  |  |  |  | a |  |
| Test de resistencias genotípicas | a | a |  | a |  | a |  | a |  | a |  | a |  | a |  | a |  | a |  |
| Sangre en EDTA (ml) | 21 | 7 | 21 | 7 | 21 | 7 | 21 | 7 | 21 |  |  |  |  |  | 21 |  |  | 21 |  |
| Sangre en ACD (ml) | 20 |  |  |  |  |  |  |  |  |  |  |  |  |  |  |  |  |  |  |

**9. Acontecimientos adversos.**

a) Información mínima a especificar:

Descripción/ definición: Acontecimiento adverso es cualquier experiencia no deseada que ocurra a un sujeto durante su participación en un ensayo clínico, se considere o no relacionada con el producto o productos en experimentación. Reacción adversa es cualquier efecto perjudicial o indeseado que se presente tras la administración de un medicamento a las dosis normalmente utilizadas en el hombre para la profilaxis, diagnóstico o tratamiento de una enfermedad.

Gravedad: Los acontecimientos graves son los que determinan el fallecimiento del sujeto, riesgo de fallecimiento, incapacidad permanente o significativa, hospitalización o prolongación de la hospitalización, o anomalías congénitas o procesos neoplásicos. (Se entiende por "riesgo de fallecimiento" la situación en que, en opinión del médico, de no haber mediado una intervención terapéutica oportuna, se hubiera producido el fallecimiento del paciente.)

Los acontecimientos adversos inesperados son aquellos que no se encuentran descritos en la información básica del producto, en cuanto a naturaleza, gravedad o frecuencia.

Método de detección y registro: los acontecimientos adversos se recogerán a partir de la cuidadosa observación clínica del paciente, análisis de laboratorio, comunicación espontánea del paciente, y también mediante un interrogatorio abierto.

De cada acontecimiento se recogerá su intensidad, duración, relación temporal con la administración del fármaco, necesidad de tratamiento y posibles causas alternativas.

b) Criterios de imputabilidad

A fin de analizar la posible relación causa-efecto, se detallará la fecha de inicio y remisión, las medidas terapéuticas adoptadas - ninguna, interrupción del tratamiento, tratamiento -, la evolución -remisión completa, secuelas, persistencia-, persistencia o no tras la suspensión de la administración, o reaparición con la readministración del producto. Para evaluar la imputabilidad el investigador usará una escala de 5 categorías.

c) Procedimientos para la notificación inmediata de los acontecimientos adversos graves o inesperados:

El investigador principal comunicará los acontecimientos adversos al Centro Coordinador, quien a su vez, informará a las autoridades sanitarias. Esta comunicación no exime al investigador de la responsabilidad de informar al Comité Ético de Investigación Clínica (CEIC) correspondiente.

Se comunicará todo acontecimiento adverso que se detecte durante el desarrollo del ensayo clínico.

Aquellos acontecimientos adversos que sean mortales, o conlleven riesgo vital, se comunicarán en el plazo de 24 horas al monitor del estudio.

Los acontecimientos adversos que, aunque no entrañen riesgo vital, sean graves o inesperados, se comunicarán en el formulario de notificación (Ver Anexo V) en el plazo de 15 días.

La información sobre acontecimientos adversos que no sean graves o inesperados, se recogerá en forma tabulada al final del ensayo clínico o coincidiendo con los análisis intermedios cuando éstos estuvieran previstos.

La información sobre acontecimientos adversos graves, recogida según las normas previas, se remitirá a la Agencia Española del Medicamento.

***

**10. Aspectos éticos.**

a) Principios generales:

El ensayo se llevará a cabo de acuerdo con los principios que emanan de la Declaración de Helsinki (Anexo IV), y según la normativa legal vigente (Real Decreto 561/1993, de 16 de abril), y no se iniciará hasta haber obtenido la aprobación del CEIC, la conformidad del Director de la Institución, y la autorización de la Agencia Española del Medicamento.

b) Información a los sujetos:

Se informará oralmente y por escrito a los los pacientes y se comunicará a los participantes toda la información pertinente adaptada a su nivel de entendimiento.

(Ver Anexo II: Hoja de información para el paciente y Anexo III: Hoja de consentimiento por escrito.)

c) Confidencialidad:

El paciente será informado de que su participación en el ensayo será tratada con la misma confidencialidad que su documentación clínica, pero que, en caso necesario, un miembro del CEIC del centro, un inspector designado por las autoridades sanitarias, o el monitor del ensayo clínico podrán tener acceso a la misma.

En el cuaderno de recogida de datos, el paciente se identificará únicamente por su número de inclusión en el estudio y sus iniciales.

El nombre del paciente no aparecerá en ninguna publicación o comunicación de los resultados del estudio.

La participación del paciente en el ensayo quedará reflejada en su historia clínica.

El investigador completará una lista en la que constarán los nombres de los pacientes que participen en el ensayo, su número de inclusión en el mismo, y su historia clínica.

d) Seguro del ensayo:

El promotor ha contratado un seguro de responsabilidad civil que cubre posibles responsabilidades por los daños derivados de la realización del presente estudio

***

**11. Consideraciones prácticas.**

a) Responsabilidades de los participantes en el ensayo:

El investigador principal revisará y aprobará el protocolo del ensayo, solicitará la autorización del CEIC, así como la conformidad del Director de la Institución, y firmará el formulario de compromiso del investigador; es responsabilidad suya, también, revisar y aprobar el informe final del ensayo clínico.

Los investigadores colaboradores tendrán la misión de evaluar la elegibilidad del paciente, informar a los pacientes y sus representantes, solicitar el consentimiento por escrito, evaluar la eficacia y seguridad del tratamiento, rellenar y firmar los Cuadernos de Recogida de Datos (CRD) y realizar el seguimiento de los acontecimientos adversos.

Los investigadores son responsables de cumplir con los requisitos del protocolo.

El monitor se asegurará de que los datos incluidos en el CRD se correspondan con los de la Historia Clínica, de que los pacientes hayan sido adecuadamente informados e incluidos en el grupo de tratamiento que les haya correspondido de manera aleatoria.

b) Condiciones de archivo, manejo, procesamiento y correcciones de datos.

El investigador principal dispondrá de un archivo con la siguiente documentación: información básica del producto, protocolo del ensayo, declaración de Helsinki, modelo de cuaderno de recogida de datos, modelo de hoja de información al paciente, hoja de consentimiento informado, formularios de notificación de acontecimientos adversos y lista de pacientes incluidos en el estudio.

1. Identificación de muestras para investigación clínica y etiquetado:

Las muestras del fármaco en estudio se utilizarán en su presentación comercial.

1. Condiciones de publicación:

Los investigadores publicarán los resultados del estudio en revistas indexadas internacionalmente.

***

**12. Análisis estadístico.**

a) Pruebas estadísticas a utilizar

- Análisis descriptivo:

Las variables referidas a los datos demográficos y antecedentes clínicos de la muestra así como las reacciones adversas se analizarán de manera descriptiva (frecuencias absolutas y relativas, media y desviación estándar, mediana y valores extremos).

*Indicaciones de tratamiento antiretroviral.*

Las recomendaciones españolas del TARV (27) indican que no se debe administrar tratamiento en los pacientes asintomáticos con una cifra de linfocitos CD4+ >500/µL y carga viral <10.000 copias/mL**.** Por tanto se determinará la proporción de estos pacientes que reune estos criterios en dos momentos: 1) cuando la CV alcance el *set-point* después de la última retirada del tratamiento; y 2) al final del estudio (semana 120-136).

- Análisis comparativo

*1) Aumento en la CV plasmática a la* ***8ª*** *semana de la retirada y 2) Proporción de pacientes con respuesta citotóxica específica frente al VIH.*

-Se compararán los ciclos 1,2,3 y 4.

-El aumento de la CV se define como un incremento de al menos 3 veces el valor basal (previo a TARV) (se corresponde aproximadamente con un aumento de 0.5 log)

-En los pacientes con una CV <10 copias/mL (indetectable), se considerará como aumento cualquier valor por encima de 20 copias/mL tras 2 semanas de interrupción del tratamiento.

-Un análisis más sensible comparará el 2º, 3º y 4º ciclos con el 1º, en cada paciente, como datos pareados. Los resultados discordantes se compararan con un test de McNemar: los N pares con un valor mas alto durante el 1º ciclo y un valor mas bajo durante 4º ciclo, con los N pares en la situación contraria. Las mismas consideraciones son aplicables a la valoración de la capacidad citotóxica y la función de los linfocitos CD4 *helper*, que se registrará como positiva vs negativa o buena vs débil según el índice de estimulación.

*2)Análisis del tiempo de incremento de la CV por encima de 5000 copias/mL.*

-El tratamiento se retirará en la semana 64-80, si la viremia en la semana 56 es menor que la de la semana 8.

-El tiempo de incremento será comparado (controles históricos) con el de otros grupos (ver referencias 2 y 3)

3) *Análisis multivariante.*

Se define como “respuesta” la ausencia de rebote o la ausencia de indicaciones para TARV en varias ocasiones después de parar el tratamiento.

Los factores predictivos de respuesta se estudiarán mediante un análisis de regresión múltiple, e incluirán:

-Recuento CD4 antes del TARV.

-CV antes de HAART.

-Respuesta citotóxica en distintos momentos del estudio.

-VIH-DNA integrado en células monocíticas en distintos momentos del estudio.

1. Análisis intermedio:

Se realizará un análisis intermedio cuando la mitad de los pacientes hayan alcanzado la semana 64-80. El estudio se terminaría en caso de que menos de 4 pacientes muestren disminución de la CV al comparar la tercera interrupción con la primera.

c) Centro de análisis:

Los datos se analizarán en la Unidad de Epidemiología y Bioestadística. Fundació Clínic.

***

1. **Bibliografía**

1. Egger M, Hirschel B, Francioli P, et al.: Impact of new anti-retroviral combination therapies in HIV-infected patients in Switzerland: prospective multicenter study. *Br Med J,* 1997, 315:1194-1195.

2. Neumann AU, Tubiara R, Calvez V, Robert C, Autran B, Katlama C: Multi-Phasic HIV Decline following Triple Drug Antiviral Therapy is Correlated with Viral Rebound Dynamics during Therapy Interruption. *5th Conference on Retroviruses,* 1998, 517.

3. Garcia F, Plana M, Vidal C, Cruceta A, Tortajada C, Gil C, Niebla C, Soriano A, Palou E, Maleno MJ, Barcelo JJ, Gallart T, Miro JM, Pumarola T, Gatell JM: Quick Viral Load Rebound After One Year Of Successful HAART In Chronic HIV-1 Infected Patients In Very Early Stages. *6th Conference on Retroviruses and Opportunistic Infections, Chicago, February 1999,* 1999, 629.

4. Chun TW, Carruth L, Finzi D, Shen XF, DiGiuseppe JA, Taylor H, Hermankova M, Chadwick K, Margolick J, Quinn TC, Kuo YH, Brookmeyer R, Zeiger MA, Barditch-Crovo P, Siliciano RF: Quantification of latent tissue reservoirs and total body viral load in HIV-1 Infection. *Nature,* 1997, 387:183-188.

5. Dyer WB, Geczy AF, Kent SJ, McIntyre LB, Blasdall SA, Learmont JC, Sullivan JS: Lymphoproliferative immune function in the Sydney Blood Bank Cohort, infected with natural nef/long terminal repeat mutants, and in other long-term survivors of transfusion-acquired HIV-1 infection. *AIDS,* 1997, 11:1565-1574.

6. Ogg GS, Jin X, Bonhoeffer S, Dunbar PR, Nowak MA, Monard S, Segal JP, Cao Y, Rowland-Jones SL, Cerundolo V, Hurley A, Markowitz M, Ho DD, Nixon DF, McMichael AJ: Quantitation of HIV-1-specific cytotoxic T lymphocytes and plasma load of viral RNA. *Science,* 1998, 279:2103-2106.

7. Greenough TC, Brettler DB, Somasundaran M, Panicali DL, Sullivan JL: Human immunodeficiency virus type 1-specific cytotoxic T lymphocytes (CTL), virus load, and CD4 T cell loss: Evidence supporting a protective role for CTL in vivo. *J Infect Dis,* 1997, 176:118-125.

8. Rosenberg ES, Billingsley JM, Caliendo AM, Boswell SL, Sax PE, Kalams SA, Walker BD: Vigorous HIV-1-specific CD4^+^ T cell responses associated with control of viremia. *Science,* 1997, 278:1447-1450.

9. Oldstone MBA: HIV versus cytotoxic T lymphocytes - The war being lost. *N Engl J Med,* 1997, 337:1306-1308.

10. Kahn JO, Walker BD: Acute human immunodeficiency virus type 1 infection. *N Engl J Med,* 1998, 339:33-39.

11. Ortiz GM, Jin X, Demoitie MA, Donahoe S, Kuebler PJ, Bonhoeffer S, Kakimoto WM, Cao Y, Ho DD, Markowitz M, Nixon DF: Containment of breakthrough HIV plasma viremia in the absence of antiretroviral drug therapy is associated with a broad and vigorous HIV specific cytotoxic T lymphocyte (CTL) response. *6th Conference on Retroviruses and Opportunistic Infections, Chicago, February 1999,* 1999, 256.

12. Lori F, Zinn D, Varga G, Seminari E, Maserati R, Miller N, Pal R, Markham P, Lisziewics J: Intermittent Drug Therapy Increases the Time to HIV Rebound in Humans and Induces the Control of SIV after Treatment Interruption in Monkeys. *6th Conference on Retroviruses and Opportunistic Infections, Chicago, February 1999,* 1999, LB5.

1. Ho DD: Toward HIV Eradication or Remission: The Tasks Ahead. *Science,* 1998, 280:1866-1867.
2. Kovacs JA, Baseler M, Dewar RJ, Vogel S, Davey RT, Falloon J et al . Increases in CD4 T Lymphocytes with Intermittent Courses of Interleukin-2 in Patients with Human Immunodeficiency Virus Infection -- A Preliminary Study. N. Engl. J. Med 1995; 332: 567.
3. De Paoli, P., S. Zanussi, C. Simonelli, M. T. Bortolin, M. D’Andrea, C. Crepaldi,R. Talamini, M. Comar, M. Giacca, and U. Tirelli. Effects of subcutaneousinterleukin-2 therapy on CD4 subsets and in vitro cytokine production in HIV 1subjects. *J. Clin. Invest.* 1997;  *100:2737.*
4. Davey, R. T., Jr., D. G. Chaitt, J. M. Albert, S. C. Piscitelli, J. A. Kovacs,R. E. Walker, J. Falloon, M. A. Polis, J. A. Metcalf, H. Masur, et al. A randomized trial of high- versus low-dose subcutaneous interleukin-2 outpatient therapy for early human immunodeficiency virus type 1 infection. *J. Infect. Dis.* 1999; *179:849.*
5. Kovacs, J. A., S. Vogel, J. M. Albert, J. Falloon, R. T. Davey, Jr., R. E. Walker,M. A. Polis, K. Spooner, J. A. Metcalf, M. Baseler, et al. Controlled trial of interleukin-2 infusions in patients infected with the human immunodeficiency virus. *N. Engl J Med.* 1996; 335:1350.
6. Zanussi S; Simonelli C; Bortolin MT; D'Andrea M; Crepaldi C; Vaccher E et al . Immunological changes in peripheral blood and in lymphoid tissue after treatment of HIV-infected subjects with highly active anti-retroviral therapy (HAART) or HAART + IL-2. *Clin Exp Immunol* 1999; 116: 486.
7. Hengge UR; Goos M; Esser S; Exner V; Dotterer H; Wiehler H et al. Randomized, controlled phase II trial of subcutaneous interleukin-2 in combination with highly active antiretroviral therapy (HAART) in HIV patients. *AIDS* 1998; 12: F225
8. Pandolfi F; Pierdominici M; Marziali M; Livia Bernardi M; Antonelli G; Galati V et al. Low-dose IL-2 reduces lymphocyte apoptosis and increases naive CD4 cells in HIV-1 patients treated with HAART. *Clin Immunol* 2000; 94: 153.
9. Chun TW, Engel D, Mizell SB, et al. Effect of interleukin-2 on the pool of latently infected resting CD4+ T cells in HIV-1-infected patients receiving highly activ antiretroviral therapy. *Nat Med* 1999; 5: 651.
10. Davey RT, Bhat N, Yoder C, et al. HIV-1 and T cell dynamics after interruption of highly active antiretroviral therapy (HAART) in patients with a history of sustained viral suppression. *Proc Natl Acad Sci USA* 1999; 96: 15109.
11. Levy Y; Capitant C; Houhou S; Carriere I; Viard JP; Goujard C et al.; Comparison of subcutaneous and intravenous interleukin-2 in asymptomatic HIV-1 infection: a randomised controlled trial. ANRS 048 study group. *Lancet*  1999; 353: 1923.
12. Bernstein ZP, Porter MM, Gould M, Lipman B, Bluman EM, Stewart CC et al. Prolonged administration of low-dose interleukin-2 in human immunodeficiency virus-associated malignancy results in selective expansion of innate immune effectors without significant clinical toxicity. *Blood* 1995; 86: 3287.
13. Jacobson EL, Pilaro F, Smith KS. Rationale interleukin 2 therapy for HIV positive individuals: daily low doses enhance immune function without toxicity. *Proc Natl Acad Sci. USA* 1996; 93: 1045.
14. Barouch DH, Craiu A, Kuroda MJ, et al. Augmentation of immune responses to HIV-1 and simian immunodeficiency virus DNA vaccines by IL-2/Ig plasmid administration in rhesus monkeys. *Proc Natl Acad Sci USA,* 2000; 97:4192.
15. Miró JM, Antela A, Arrizabalaga J, Clotet B, Gatell JM, Guerra L, Iribarren JA, Laguna F, Moreno S, Parras F, Rubio R, Santamaría JM, Viciana P por el Grupo de Estudio de Sida (GESIDA) y por Consejo Asesor Clínico (CAC) de la Secretaría Plan Nacional sobre el Sida (SPNS) del Ministerio de Sanidad y Consumo (MSC). Recomendaciones de GESIDA/Plan Nacional sobre el Sida respecto al tratamiento antirretroviral en pacientes adultos infectados por el vih en el año 2000*. www.gesidaseimc.com*. Edición de junio del 2000.

***

1. El primer ciclo va de la semana 0 a la 10, empezando por las 2 semanas sin tratamiento. La viremia en las semanas 9, 19, 29 y 39 determinará cuando se podrá volver a parar el tratamiento en el siguiente ciclo, solo los pacientes en los que la vieremia es <50 copias/mL en la semana 9 pararán el tratamiento a la semana 10, etc. [↑](#footnote-ref-2)
2. determinación de A2 y B25. Solo los pacientes con los 2 antígenos (75% de la población) pueden tomar parte; la presencia de A2 o B25 es un requisito para medir la respuesta citotóxica gag-específica . [↑](#footnote-ref-3)
3. En pacientes con HLA35 o A2 (70% de los pacientes)

   ^4^ Biopsia amigdalar. [↑](#footnote-ref-4)
