# Supplementary material for: Structured Treatment Interruptions and Low Doses of IL-2 in Patients with Primary HIV Infection. Inflammatory, Virological and Immunological Outcomes
Source: PLoS One. 2015 Jul 17;10(7):e0131651. doi: 10.1371/journal.pone.0131651 (PMC4506046; doi:10.1371/journal.pone.0131651)
Supplement: S2 Protocol — (DOC) [file pone.0131651.s003.doc]

*CLINICAL MEDICATION TRIAL PROTOCOL:*

| PILOT STUDY TO EVALUATE INTERMITTENT THERAPY AND INTERLEUKIN-2 IN HIV POSITIVE PATIENTS TREATED DURING PRIMARY INFECTION |
| --- |

Protocol: PHI-IL2

Version: 1.0 (13/09/2001)

Infections Service

Hospital Clínic,

Villarroel, 170,

08036 Barcelona

Tel. 93 2275430

Fax: 93 4514438

**1. Summary**

1.0. Type of request:

Clinical trial with a new indication.

1.1. Researchers:

| Dr. José Mª Miró Meda  Infectious Diseases Service  Hospital Clínic,  Villarroel, 170,  08036 Barcelona | Dr. José Mª Gatell Artigas  Infectious Diseases Service  Hospital Clínic,  Villarroel, 170,  08036 Barcelona |
| --- | --- |

1.2. Title:

PILOT STUDY TO EVALUATE INTERMITTENT THERAPY AND INTERLEUKIN-2 IN HIV POSITIVE PATIENTS TREATED DURING PRIMARY INFECTION

1.3. Protocol code:

PHI-IL2

1.4. Principal researchers:

| Dr. José Mª Miró Meda  Infectious Diseases Service  Hospital Clínic,  Villarroel, 170,  08036 Barcelona | Dr. José Mª Gatell Artigas  Infectious Diseases Service  Hospital Clínic,  Villarroel, 170,  08036 Barcelona |
| --- | --- |

1.5. Planned Test Centres:

Institute of Infectious Diseases and Clinical Immunology

Hospital Clínic,

Villarroel, 170,

08036 Barcelona

1.6. Ethical approval by clinical research commissions:

The test has been submitted for approval to the clinical research ethics committee, the CEIC, of the Corporació Sanitària Clínic.

1. Supervision:

Dr. Anna Cruceta Arbolés

Infections Service

Hospital Clínic,

Barcelona

1.8. Experimental drug:

Aldesleukin (recombinant Interleukin-2) (IL-2) Proleukin®, Chiron

18 mUI vials

Therapeutic group: L01F1A

1.9. Phase of the trial: Phase III

1.10. Primary goal:

To evaluate the virological response, the specific cytotoxic response to HIV and the point of stabilisation of viremia after the fourth cycle of interruption of anti-retroviral treatment (ART), and the proportion of patients who do not require ART at the end of study following the administration of subcutaneous, low doses of IL-2.

1.11. Experimental design:

A single-centre, non-comparative, prospective, open-label, clinical pilot study.

1.12. Disease being studied:

HIV-1 infection.

1.13. Main evaluation variables:

The proportion of patients presenting plasma viral load (VL) three times greater than the base value (prior to ART), or VL >20 copies/mL where base VL <10 copies/mL;

The proportion of patients with good cytotoxic response after the fourth cycle compared with the beginning of the treatment;

The proportion of patients having been administered with IL-2 not requiring ART at the end of the study. (>350 CD4 cells or VL <55,000 copies/mL).

1.14. Test population:

The study will include eight HIV infected, outpatients having begun highly active antiretroviral treatment (HAART) during primary infection.

1. Duration of the treatment:

The treatment will be administered daily from the first interruption of ART with a subcutaneous dose of 125,000 mIU/m^2^ for six weeks. After completing four cycles of six weeks, the treatment with IL-2 will be stopped. Patient follow-up will be conducted for two years.

1.16. Schedule and anticipated date of conclusion:

The aim is to start the trial, provided authorisation has been obtained, in November 2001. The recruitment period will last two months, and the follow-up will last two years. The aim is to finish the study in December 2003.

***

**2. Contents** page

1. Summary 2

2. Contents 5

3. General information 6

4. Rationale and objectives 8

5. Trial type and design 12

6. Subject selection 13

7. Description of the treatment 15

8. Trial development and response evaluation 17

9. Adverse events 19

10. Ethical aspects 21

11. Practical considerations 22

12. Statistical analysis 23

13. References 25

Annexes:

Annex I Data collection notebook (DCN)

Annex II Patient information sheet

Annex III Informed consent form

Annex IV Declaration of Helsinki

Annex V Notification of adverse events sheet

Annex VI Civil liability insurance

Annex VII Information on trial products

**3. General information**

A. Trial identification

1st Code: PHI-IL2

2nd Title: PILOT STUDY TO EVALUATE INTERMITTENT THERAPY AND INTERLEUKIN-2 IN HIV POSITIVE PATIENTS TREATED DURING PRIMARY INFECTION

B. Type of clinical trial:

Clinical trial with a new indication

C. Description of trial drugs:

Generic denomination, commercial name, pharmaceutical form and composition:

Aldesleukin (recombinant Interleukin-2) (IL-2) Proleukin®, Chiron

18 mUI vials

D. Researchers:

| Dr. José Mª Gatell Artigas  Infectious Diseases Service  Hospital Clínic,  Villarroel, 170,  08036 Barcelona | Dr. José Mª Miró Meda  Infectious Diseases Service  Hospital Clínic,  Villarroel, 170,  08036 Barcelona |
| --- | --- |

Telephone: 93 227 54 00 Fax: 93 451 44 38

E. Technician in charge of producing samples:

Technician at the manufacturing laboratory (Chiron)

F. Supervisor:

Dr. Anna Cruceta Arbolés

Infections Service

Hospital Clínic,

Barcelona

G. Principal researchers:

| Dr. José Mª Miró Meda  Infectious Diseases Service  Hospital Clínic,  Villarroel, 170,  08036 Barcelona | Dr. José Mª Gatell Artigas  Infections Service  Hospital Clínic,  Villarroel, 170,  08036 Barcelona |
| --- | --- |

H. Ethical centres and commissions:

The test has been submitted for approval to the clinical research ethics committee, the CEIC, of the Corporació Sanitària Clínic.

1. Anticipated duration of the test:

The aim is to start the trial, provided authorisation has been obtained, in November 2001. The recruitment period will last two months, and the follow-up will last two years. The aim is to finish the study in December 2003.

***

**4. Rationale and objectives**

a) *Specific immunity to HIV and intermittent therapy*

Highly active anti-retroviral therapy (HAART) has reduced to the incidence of AIDS-defining diseases by approximately 85% [1]. Nevertheless it entails an indefinite treatment with at least three drugs and therefore implies discomfort, undesirable side-effects and high cost. Until now, attempts to stop anti-retroviral treatment (ART) have failed. Even in patients whose VL has remained undetectable for months, withdrawal from treatment has invariably been followed by rebound in VL within a few weeks [2,3]. The rebound of VL probably begins in reservoirs in latently-infected lymphocytes [4].

Although HIV destroys the immune system in most patients, a small group of patients known as long-term non-progressors (LTNP) apparently tolerate the infection for many years with few effects. In these patients, CD4 cells stay within the normal range, and VL is very low or undetectable. Most LTNPs have good and persistent lymphocytic cytotoxic response to HIV [5-8]. Nevertheless this response is transitory in non-LTNP patients, allowing the continued proliferation of the HIV infection that leads eventually to immunosuppression [9].

Preliminary data indicate that it could be possible to induce cytotoxic T cells (CTL) in patients who otherwise would progress towards immunodeficiency and that these patients control viral proliferation after stopping treatment:

- Patients treated very promptly post-infection (during the primary infection or shortly after) develop a powerful and lasting cytotoxic response to HIV [10].

- One case study reports of two patients with bad adherence to therapy who stopped treatment, causing a rebound in VL. They later resumed treatment for different lengths of time before finally withdrawing from treatment indefinitely. In both cases the VL remained undetectable for 14 and 21 months without ART. In both cases, patients presented a good and ample cytotoxic response to HIV that was probably strengthened by the rebound in VL during the first interaction in treatment. Cytotoxic response was evaluated whilst the patient was out of treatment, and it continued to remain high [11].

- Another patient treated with DDI, hydroxyurea and indinavir stopped the treatment and presented a rebound in viremia. The patient went back into treatment, after which VL decreased; treatment was then stopped again. Viremia did not rebound and remained at the same level for more than one year without treatment. The patient presented good cytotoxic response to HIV [12].

- Three patients with stable viremia were treated with a combination containing hydroxyurea under the following schedule: three weeks of treatment, one week of interruption followed by two cycles of three months therapy with interruption and resumption of treatment once VL went back up (>5,000 copies/mL). VL became undetectable (<400 copies/mL) after each return to treatment. The relapse-free intervals increased by seven days after the first interruption and went up to 37 days after the third interruption [12].

-*Rhesus macaques* infected with SIV/Mac251 (basal viremia: 200,000 copies/mL, 500,000 copies/mL and 1.1 million copies/mL) were treated with hydroxyurea, DDI and a derivative of adefovir following an intermittent schedule (two cycles of three weeks of treatment followed by interruption). SIV was allowed to rebound to >5,000 copies/mL. Although VL rebounded, in three cases it was controlled and stabilised to a low level of viremia in the absence of treatment. This level was >5,000 copies/mL and was maintained for six weeks during the first interruption and reduced to <200 copies/mL at the lowest and maintained for ten weeks during the third interruption. Two of the monkeys continued without relapse after four and six months respectively [12].

The intermittent interruption of treatment is associated with a strengthening of the cytotoxic response to HIV that can contribute to a prolonged suppression of VL when treatment is withdrawn.

1. *Acute suspension of treatment is acceptably safe*

Chronic exposure to sub-therapeutic levels of anti-retrovirals can promote resistant mutations and lead to a loss in efficacy. Nevertheless the abrupt interruption of treatment does not seem to be associated with the formation of resistance. In the cases of interruption of the treatment, viral proliferation originates in viral reservoirs in non-activated T lymphocytes. Numerous studies have demonstrated that the virus remaining in the reservoirs is sensitive to ART [13].

Neumann et al. and Garcia et al. have reported triple treatment interruption therapies, with follow-up in patients until VL rebounded and reached stable levels, before returning to ART. In the 23 patients, control of VL was at least as quick during the second round of treatment as it was in the first round. All patients had a VL of <200 copies/mL at six weeks of the second course of treatment.

1. *Effects of IL-2 administration*

Numerous studies have looked at combining ART with three or more anti-retroviral drugs with cycles of IL-2 administered during five days in high (18 million UI/day) or intermediate (3-6 million UI/day) doses via i.v. or s.c. every eight weeks (14-23). This approach has been used in advanced patients (with CD4+ lymphocyte counts <200-500 cell/µL) with the aim of increasing the number of CD4+ lymphocytes, improving other immunological parameters and reducing the apoptosis of T lymphocytes. In addition, observations have shown that the concurrent administration of elevated doses of IL-2 and ART reduces the pool of latent CD4+ lymphocytes that contain the DNA of integrated HIV-1 (21). Nevertheless, when ART is stopped, there is a fast rebound of HIV-1 plasma VL (22). On the other hand, high or intermediate IL-2 doses have numerous side-effects, mainly when administered by i.v. (24). This toxicity is dose-dependent. There are no adverse effects when it is administered in low doses. In this sense, exceptional adverse effects were noted in a study of patients with neoplasia related to HIV-1 infection receiving low doses of IL-2 (0.5-1.2 million UI/day) for 3 months but, on the other hand, there were no observations of opportunistic infections or increases in HIV-1 plasma VL, embodying an immunological improvement of Th1 cell response (24).

The aim of administering very low doses of IL-2 to patients with primary HIV-1 infection treated from the beginning of the acute infection is based on a study by the group of Dr. KA Smith. In this study, very low doses of IL-2 were administered during six months via s.c. in asymptomatic patients with a chronic HIV-1 infection receiving single-agent ART and having a relatively high HIV-1 plasma VL. The patients did not suffer side-effects and were able to self-administer IL-2 and to maintain daily activities (31). In addition, it was shown that with this dose of IL-2, a series of immunological benefits were to be obtained: 1) a qualitative improvement in the immune system, measured by delayed hypersensitivity antigen skin memory tests; 2) an increase in CD4+ lymphocytes; 3) an absence of significant changes in HIV-1 plasma VL; and 4) an absence of measurable increases of pro-inflammatory plasma cytokines (TNF-α, IFN-γ, GM-CSF) (31). The maximum non-toxic therapeutic dose over six months was obtained with the daily s.c. administration of 187,000-250,000 unit/m^2^/day. Peak plasma IL-2 levels were 22 pM, sufficient to saturate ≥70% of high affinity IL-2 receivers in T, B and NK cells, but only 2% of low affinity IL-2 receivers. This study also demonstrated the effectiveness of the administration of a single dose of 125,000 units/m^2^/day (25). So it seems this very low, side-effect-free IL-2 dose is an ideal addition to ART to expand selectively *in vivo* high affinity IL-2 receivers in CD4+ and CD8+ lymphocytes once activated with a specific antigenic stimulus, in this case, HIV-1 during the period of ART interruption. This is possible as CD4+ lymphocytes have T-cell receivers capable of recognising the antigens in the antigen-presenting cells. On the basis of this data, we think that combining IL-2 with structured interruptions of ART could be feasible and could expand the specific HIV response of CD4+ and CD8+ lymphocytes once activated during the rebound of VL and to allow a control of the HIV-1 viral replication after the suspension of ART after the fourth cycle. This hypothesis was recently verified in an experiment on higher primates, in which the administration of IL-2-Ig, which is functionally more efficient than the natural IL-2, significantly increased the cellular and humoral immune response of animals vaccinated with HIV-1 and SIV DNA (26).

General aim:

To evaluate the virological response and the specific cytotoxic response to HIV, the point of stabilisation of viremia after fourth cycle of interruption of anti-retroviral treatment (ART), and the proportion of patients who do not require ART at the end of study following the administration of subcutaneous, low doses of IL-2.

Specific aims:

- To determine the proportion of patients with VL increases at least three times greater than the baseline value (prior to ART) (corresponding approximately to an increase of 0.5 log).
- To determine the proportion of patients with specific cytotoxic response to HIV
- Compare the level of viremia stabilisation at the point of stopping ART by comparing viremia at the end of the fourth cycle and at the end of the study with pre-HAART viremia.
- Determine the proportion of patients not requiring ART at the end of the study.

***

**5. Trial type and design.**

1. Phase of development:

Phase III

1. Description of the randomisation process:

Not applicable as the trial is non-comparative.

c) Type of control and design:

A single-centre, non-comparative, prospective, open-label, clinical pilot study.

d) Blinding methods:

Not applicable as this is an open-label trial.

e) Follow-up period:

Two years

***

**6. Subject selection**

a) Inclusion criteria:

The trial will include patients of both sexes:

1. with diagnosed HIV-1 infection;
2. aged 18 years or older;
3. treated with HAART anti-retroviral treatment (composed of two analogues non-nucleoside reverse transcriptase inhibitors plus a protease inhibitor) since primary infection;
4. plasma viral load <50 copies/mL (Roche Monitor Assay) for at least eight months;
5. CD4 lymphocyte count consistently >500 cells/μL (prior to stopping treatment); and
6. who, having been appropriately informed, have granted written consent to take part in the study and to be subjected to tests and examinations that it entails.

b) Exclusion criteria:

1. Patients who have responded suitably to the initial anti-retroviral treatment;
2. patients who have at some time taken nevirapine or efavirenz; and
3. women who are pregnant or lactating, or who intend to become pregnant during the period of the study.

c) Predicted number of subjects:

There will be eight outpatients.

d) Methods for calculating the sample size and data used:

As this is a pilot study, there is no way of knowing what kind of data will be produced in response to this treatment in this subtype of patients with primary HIV infection. The results of this study will allow the calculation of the sample necessary to create a comparison with an appropriate statistical basis.

e) Discontinuation criteria and anticipated analysis of withdrawals and discontinuations:

Patients will be able to leave the study at any time without having to give explanations. Nevertheless, the causes for discontinuation will be collected and recorded in the data collection notebook (DCN).

The researcher will also be able to withdraw patients from the study for reasons of inefficiency, adverse events or patient benefit. The reason for withdrawal will be recorded in the DCN.

All patients who, having been assigned to a treatment group, discontinue the treatment under study or who are withdrawn for any cause will be included in an “intention-to-treat” analysis.

The patients who fail to attend appointments (loss of follow-up) will be considered for the analysis until the moment at which the loss takes place.

f) Dealing with pre-randomisation losses:

Patients who fulfil selection criteria but who, for whatever reason, are not included in one of the treatment groups will be recorded as appropriate (personal, demographic data, etc.), but they will not be included in the analysis.

g) Approximate duration of enrolment:

A period of two months is planned to recruit eight patients with the described characteristics.

***

**7. Description of the treatment**

Patients will stop antiretroviral treatment for eight weeks and will return to it for eight to twelve additional weeks with the addition of interleukin-2 for six weeks.

This process will be repeated four times. Upon finishing the four cycles, treatment with IL-2 and ART will be suspended unless there has been evidence of viral replication to the point that viral load (VL) reaches a stable level. (Definition of point stabilization: equal VL in three sequential determinations separated by at least three weeks)

a) Doses, intervals, method and form of administration and duration

Interleukin-2 (IL-2), 125,000 units/m^2^/day, subcutaneously, for six weeks

b) Criteria for modifying the protocol during the trial:

• INTOLERANCE/TOXICITY

Interleukin-2: the tolerance of the medication will be evaluated at each visit with a view to its suspension in cases where the severity or intensity of adverse effects so prescribe.

• SAFETY MEASURES

Because of concerns over an eventual appearance of resistance and a loss of antiretroviral treatment efficacy, various measures have been designed to ensure that intermittent therapy will be immediately discontinued in case there is a failure to:

- Analyse the response to treatment in the weeks leading up to each interruption. VL will be measured at seven weeks after restarting treatment in each cycle. The study will be stopped if more than two favourable responding patients reach VL <50 copies/mL.

- The study will only be interrupted between weeks 64 to 80 in those patients who show lowered VL after the third interruption compared with the first interruption.

- ART will be started in patients with CD4+ lymphocyte count <350 cells/µL or with an HIV-1 plasma VL >55,000 copies/mL in two consecutive determinations (27). In asymptomatic patients with CD4+ lymphocyte count between 350-500 cells/µL and/or VL between the previous levels, the evaluation of resuming ART will be done on an individual basis.

- There will be a safety commission.

c) Concomitant medications:

Any concomitant medication will be reflected appropriately in the DCN (detailing the product, dose, administration method, day of administration, reason for the treatment, etc.).

1. Salvage medication:

ART will be started in patients with CD4+ lymphocyte count <350 cells/µL or with an HIV-1 plasma VL >55,000 copies/mL in two consecutive determinations (27).

The salvage medication will also be appropriately recorded in the DCN throughout the entire follow-up period of the trial.

1. Standards for handling trial drugs:

The pharmaceutical specialties used in the study do not require special conditions for conservation or handling, under normal conditions of humidity and temperature.

***

**8. Trial development and response evaluation**

a) Main evaluation variables (activity and effectiveness):

- To determine the proportion of patients with VL increases at least three times greater than the baseline (prior to ART) (corresponding approximately to an increase of 0.5 log).
- To determine the proportion of patients with specific cytotoxic response to HIV
- Compare the level of viremia stabilisation at stopping ART by comparing viremia at the end of the fourth cycle and at the end of the study with pre-HAART viremia.
- Determine the proportion of patients not requiring ART at the end of the study.

b) Secondary variables (safety and tolerance):

- Description of adverse events

c) Number and timing of visits and tests or examinations.

Medical visits will be carried out during the first week of the study, then every fortnight for four months and then every two months until the end of the study. During the periods of treatment interruption analyses will be carried out weekly. During the periods of treatment the analyses will be monthly.

An amygdala biopsy will be taken at the beginning and at the end of the study

See calendar (p. 18).

d) Duration of the study:

Two and a half years from the date of the first patient inclusion. The recruitment period should be two months and the duration of the study 24 months from the date of the last patient inclusion.

Calendar of the visits and tests or examinations.

| Determination | 0 | 8 | 16[[1]](#footnote-2) | 24 | 32 | 40 | 48 | 56 | 64 | 72 | 80 | 88 | 96 | 104 | 112 | 120 | 128 | 136 | >136 |
| --- | --- | --- | --- | --- | --- | --- | --- | --- | --- | --- | --- | --- | --- | --- | --- | --- | --- | --- | --- |
| VL (limit of detectability = 10) | a | a | a | a | a | a | a | a | a | a | a | a | a | a | a | a | a | a | Every 4 weeks |
| Complete blood count | a |  |  |  |  |  |  |  | a |  |  |  |  |  | a |  |  | a | Every 8 weeks |
| CD4 | a |  |  |  |  |  |  |  | a |  |  |  |  |  | a |  |  | a | Every 8 weeks |
| Histocompatibility[[2]](#footnote-3) | a |  |  |  |  |  |  |  |  |  |  |  |  |  |  |  |  |  |  |
| Cytotoxic function in response to HIV and CD4 helper function[[3]](#footnote-4) | a |  | a |  | a |  | a |  | a |  |  |  |  |  | a |  |  |  | Every 16 weeks |
| Integrated HIV DNA  In peripheral mononuclear cells (PMC) | a |  | a |  | a |  | a |  | a |  |  |  |  |  | a |  |  |  | Every 16 weeks |
| Plasma and PMC conservation | a |  | a |  | a |  | a |  | a |  |  |  |  |  | a |  |  | a | Every 8 weeks |
| Lymphoid tissue[[4]](#footnote-5) | a |  |  |  |  |  |  |  |  |  |  |  |  |  |  |  |  | a |  |
| Genotype resistance test | a | a |  | a |  | a |  | a |  | a |  | a |  | a |  | a |  | a |  |
| Blood in EDTA (ml) | 21 | 7 | 21 | 7 | 21 | 7 | 21 | 7 | 21 |  |  |  |  |  | 21 |  |  | 21 |  |
| Blood in ACD (ml) | 20 |  |  |  |  |  |  |  |  |  |  |  |  |  |  |  |  |  |  |

**9. Adverse events**

a) Reporting standards:

Description/definition: An adverse event is any undesired effect on a subject during their participation in a clinical trial and which is considered unrelated to the drug or drugs being studied. An adverse reaction is any detrimental and desired effect that occurs following the administration of a medication in the normal doses in a human being for prophylaxis, diagnosis or treatment of an illness.

Severity: Serious adverse events are those that lead to the death of a subject, the risk of death, persistent or significant disability, hospitalisation or the extension of hospitalisation, or congenital anomalies or neoplasia. (A situation involving “the risk of death” is one in which, in the doctor’s opinion, failing to have taken timely therapeutic intervention would have led to the death of the patient.)

Unexpected adverse events are those that are not described in the basic information of the product in terms of nature, severity or frequency.

Detection and registration method: adverse events will be monitored via careful clinical observation of the patient, laboratory analysis, spontaneous communication with the patient, and through open questioning.

Each event will be recorded in terms of its intensity, duration, the relation to the timing of the drug administration, the need for treatment and alternative possible causes.

b) Attribution criteria

With a view to analysing the possible cause and effect relationships, the following will be recorded: start and remission date, therapeutic measures adopted (none, interruption of treatment, treatment), evolution (full remission, after effects, persistence), persistence or otherwise following suspension of administration, or the reappearance with fresh administration of the drug. With a view to evaluating the attribution of the researcher, a five-point scale will be used.

c) Procedures for the immediate notification of serious or unexpected adverse events:

The main researcher will inform the coordinating centre of adverse events, and the latter will inform the health authorities. By informing the coordinating centre, the main investigator will not be relieved from the duty of informing the corresponding clinical research ethics commission (CEIC).

All adverse events that are detected throughout any period of the clinical trial will be communicated.

Any adverse event that leads to death, or that carries the risk of death, will be communicated to the trial supervisor within 24 hours.

Any adverse events that, whilst not carrying the risk of death, are serious or unexpected will be communicated via the notification form (see Annex V) within two weeks.

Any information relating to adverse events that are neither serious nor unexpected will be recorded in tables at the end of the clinical trial or at an interim analysis where applicable.

Information on serious adverse events, recorded in compliance with the *ex-ante* standards, will be submitted to the national medication agency (Agencia Española del Medicamento).

***

**10. Ethical aspects**

a) General principles:

The trial will be carried out in compliance with the principles enshrined in the Declaration of Helsinki (Annex IV), and in compliance with current legal standards (Royal Decree 561/1993 of 16 April), and will not begin until the appropriate approval/authorisation has been granted by the CEIC, the Institute’s Director, and the national medication agency (Agencia Española del Medicamento).

b) Informing subjects:

Patients will be informed orally and in writing, and all participants will receive all of the relevant information adapted to their level of understanding.

(See Annex II: Patient information sheet, and Annex III: Informed consent form).

c) Confidentiality:

The patient will be informed that their participation in the trial will be treated with the same confidentiality as their clinical documentation, but that, where necessary, access may be granted to a member of the CEIC of the centre, a designated health authority inspector, or the clinical trial supervisor.

In the DCN, patients will only be identified by their initials and their patient inclusion number for the study.

The name of the patient will not appear in any publication or communication of the results of the study.

The participation of the patient in the test will be reflected in his/her clinical history.

The investigator will compile a list containing the names of patients taking part in the trial, their inclusion number, and their clinical history.

d) Trial safety:

The trial promoter has taken out a civil liability insurance policy that covers all liabilities for damage entailed by carrying out the current trial.

***

**11. Practical considerations**

a) Responsibilities of participants:

The main researcher will review and approve the trial protocol, request authorisation from the CEIC, as well as the agreement of the Director of the Institute, sign the research commitment form; the main researcher also bears the responsibility for reviewing and adopting the final clinical trial report.

Other members of the research team will undertake the patient eligibility evaluation, inform patients and their representatives, request written consent, evaluate the efficacy and safety of treatment, complete and sign the CDN and carry out follow-up of adverse events.

The researchers are responsible for fulfilling the requirements of the protocol.

The supervisor will ensure that data included in the CDN corresponds with the data in the clinical history and that patients have been appropriately informed and included in the treatment group to which they were assigned through a random means.

b) Storage, handling, processing and correcting data:

The main researcher will possess a file containing the following information: basic drug information, trial protocol, the Declaration of Helsinki, CDM template, patient information sheet template, informed consent form, adverse events notification form and a list of patients included in the trial.

1. Identification and labelling of samples for clinical trials:

The samples of the drug under investigation will be used in their commercial presentation.

1. Publication:

Investigators will publish the results of the study in internationally indexed journals.

***

**12. Statistical analysis**

a) Statistical tests:

- Descriptive analysis:

The descriptive analysis will include variables referring to demographic data and clinical events related to the sample as well as adverse reactions in terms of absolute and relative frequency, means and standard deviations, medians and ranges.

*Indications for antiretroviral treatment*

Spanish recommendations for ART (27) prescribe that it should not be given to patients who are asymptomatic with CD4+ lymphocyte cell count <500 µg/L and a viral load <10,000 copies/mL. The proportion of patients presenting both criteria will be determined at two points: 1) when VL reaches the set-point following the last withdrawal from treatment; and 2) at the end of the study (week 120-136).

- Comparative Analysis

*1) Increase in plasma VL in the* ***eighth*** *week following withdrawal, and 2) proportion of patients with cytotoxic response to HIV.*

- Comparison will be made between cycles one, two, three and four.

An increase in VL is defined as being at least three times greater than the baseline (prior to ART) (corresponding approximately to an increase of 0.5 log).

- In patients with a VL <10 copies/mL (undetectable), an increase will be considered as being any value >20 copies/mL after two weeks since the interruption of treatment.

- A more sensitive analysis will compare the second, third and fourth cycles with the first cycle, for each patient, as paired data. McNemar’s test will be used to compare discordant results: i.e., N pairs with a higher value for the first cycle and a lower value for the fourth cycle, with the N pairs from the opposite situation. The same considerations will be applied to the evaluation of the cytotoxic capacity and function of CD4 *helper* lymphocytes, which will be recorded as positive vs. negative or good vs. weak versus according to the stimulation index.

*2) Analysis of VL increase time above 5,000 copies/mL.*

- The treatment will be stopped in weeks 64-80, if viremia in week 56 is less than in week eight.

- The increase time will be compared (measurement history) with that of other groups (see references 2 and 3)

3) *Multivariant analysis*

A ‘response’ is defined as the lack of a rebound or the absence of indications for ART on multiple occasions following the interruption of treatment.

The indicating response factors will be evaluated by means of multiple regression analysis, and will include:

- CD4 count before ART,

- VL before HAART,

- Cytotoxic response at various points in the study,

- integrated HIV-DNA in monocytes at various points in the study.

1. Interim analysis:

An interim analysis will be conducted when half of the patients have reached weeks 64-80. The study will be terminated if less than four patients show reduced VL between the third and the first interruption.

c) Analysis centre:

Data will be analysed at the Epidemiology and Biostatistics Unit, Fundació Clínic.

***

1. **References**

1. Egger M, Hirschel B, Francioli P, et al.: Impact of new anti-retroviral combination therapies in HIV-infected patients in Switzerland: prospective multicenter study. *Br Med J,* 1997, 315:1194-1195.

2. Neumann AU, Tubiara R, Calvez V, Robert C, Autran B, Katlama C: Multi-Phasic HIV Decline following Triple Drug Antiviral Therapy is Correlated with Viral Rebound Dynamics during Therapy Interruption. *5th Conference on Retroviruses,* 1998, 517.

3. Garcia F, Plana M, Vidal C, Cruceta A, Tortajada C, Gil C, Niebla C, Soriano A, Palou E, Maleno MJ, Barcelo JJ, Gallart T, Miro JM, Pumarola T, Gatell JM: Quick Viral Load Rebound After One Year Of Successful HAART In Chronic HIV-1 Infected Patients In Very Early Stages. *6th Conference on Retroviruses and Opportunistic Infections, Chicago, February 1999,* 1999, 629.

4. Chun TW, Carruth L, Finzi D, Shen XF, DiGiuseppe JA, Taylor H, Hermankova M, Chadwick K, Margolick J, Quinn TC, Kuo YH, Brookmeyer R, Zeiger MA, Barditch-Crovo P, Siliciano RF: Quantification of latent tissue reservoirs and total body viral load in HIV-1 Infection. *Nature,* 1997, 387:183-188.

5. Dyer WB, Geczy AF, Kent SJ, McIntyre LB, Blasdall SA, Learmont JC, Sullivan JS: Lymphoproliferative immune function in the Sydney Blood Bank Cohort, infected with natural nef/long terminal repeat mutants, and in other long-term survivors of transfusion-acquired HIV-1 infection. *AIDS,* 1997, 11:1565-1574.

6. Ogg GS, Jin X, Bonhoeffer S, Dunbar PR, Nowak MA, Monard S, Segal JP, Cao Y, Rowland-Jones SL, Cerundolo V, Hurley A, Markowitz M, Ho DD, Nixon DF, McMichael AJ: Quantitation of HIV-1-specific cytotoxic T lymphocytes and plasma load of viral RNA. *Science,* 1998, 279:2103-2106.

7. Greenough TC, Brettler DB, Somasundaran M, Panicali DL, Sullivan JL: Human immunodeficiency virus type 1-specific cytotoxic T lymphocytes (CTL), virus load, and CD4 T cell loss: Evidence supporting a protective role for CTL in vivo. *J Infect Dis,* 1997, 176:118-125.

8. Rosenberg ES, Billingsley JM, Caliendo AM, Boswell SL, Sax PE, Kalams SA, Walker BD: Vigorous HIV-1-specific CD4^+^ T cell responses associated with control of viremia. *Science,* 1997, 278:1447-1450.

9. Oldstone MBA: HIV versus cytotoxic T lymphocytes - The war being lost. *N Engl J Med,* 1997, 337:1306-1308.

10. Kahn JO, Walker BD: Acute human immunodeficiency virus type 1 infection. *N Engl J Med,* 1998, 339:33-39.

11. Ortiz GM, Jin X, Demoitie MA, Donahoe S, Kuebler PJ, Bonhoeffer S, Kakimoto WM, Cao Y, Ho DD, Markowitz M, Nixon DF: Containment of breakthrough HIV plasma viremia in the absence of antiretroviral drug therapy is associated with a broad and vigorous HIV specific cytotoxic T lymphocyte (CTL) response. *6th Conference on Retroviruses and Opportunistic Infections, Chicago, February 1999,* 1999, 256.

12. Lori F, Zinn D, Varga G, Seminari E, Maserati R, Miller N, Pal R, Markham P, Lisziewics J: Intermittent Drug Therapy Increases the Time to HIV Rebound in Humans and Induces the Control of SIV after Treatment Interruption in Monkeys. *6th Conference on Retroviruses and Opportunistic Infections, Chicago, February 1999,* 1999, LB5.

1. Ho DD: Toward HIV Eradication or Remission: The Tasks Ahead. *Science,* 1998, 280:1866-1867.
2. Kovacs JA, Baseler M, Dewar RJ, Vogel S, Davey RT, Falloon J et al . Increases in CD4 T Lymphocytes with Intermittent Courses of Interleukin-2 in Patients with Human Immunodeficiency Virus Infection -- A Preliminary Study. N. Engl. J. Med 1995; 332: 567.
3. De Paoli, P., S. Zanussi, C. Simonelli, M. T. Bortolin, M. D’Andrea, C. Crepaldi,R. Talamini, M. Comar, M. Giacca, and U. Tirelli. Effects of subcutaneousinterleukin-2 therapy on CD4 subsets and in vitro cytokine production in HIV 1subjects. *J. Clin. Invest.* 1997;  *100:2737.*
4. Davey, R. T., Jr., D. G. Chaitt, J. M. Albert, S. C. Piscitelli, J. A. Kovacs,R. E. Walker, J. Falloon, M. A. Polis, J. A. Metcalf, H. Masur, et al. A randomized trial of high- versus low-dose subcutaneous interleukin-2 outpatient therapy for early human immunodeficiency virus type 1 infection. *J. Infect. Dis.* 1999; *179:849.*
5. Kovacs, J. A., S. Vogel, J. M. Albert, J. Falloon, R. T. Davey, Jr., R. E. Walker,M. A. Polis, K. Spooner, J. A. Metcalf, M. Baseler, et al. Controlled trial of interleukin-2 infusions in patients infected with the human immunodeficiency virus. *N. Engl J Med.* 1996; 335:1350.
6. Zanussi S; Simonelli C; Bortolin MT; D’Andrea M; Crepaldi C; Vaccher E et al . Immunological changes in peripheral blood and in lymphoid tissue after treatment of HIV-infected subjects with highly active anti-retroviral therapy (HAART) or HAART + IL-2. *Clin Exp Immunol* 1999; 116: 486.
7. Hengge UR; Goos M; Esser S; Exner V; Dotterer H; Wiehler H et al. Randomized, controlled phase II trial of subcutaneous interleukin-2 in combination with highly active antiretroviral therapy (HAART) in HIV patients. *AIDS* 1998; 12: F225
8. Pandolfi F; Pierdominici M; Marziali M; Livia Bernardi M; Antonelli G; Galati V et al. Low-dose IL-2 reduces lymphocyte apoptosis and increases naive CD4 cells in HIV-1 patients treated with HAART. *Clin Immunol* 2000; 94: 153.
9. Chun TW, Engel D, Mizell SB, et al. Effect of interleukin-2 on the pool of latently infected resting CD4+ T cells in HIV-1-infected patients receiving highly activ antiretroviral therapy. *Nat Med* 1999; 5: 651.
10. Davey RT, Bhat N, Yoder C, et al. HIV-1 and T cell dynamics after interruption of highly active antiretroviral therapy (HAART) in patients with a history of sustained viral suppression. *Proc Natl Acad Sci USA* 1999; 96: 15109.
11. Levy Y; Capitant C; Houhou S; Carriere I; Viard JP; Goujard C et al.; Comparison of subcutaneous and intravenous interleukin-2 in asymptomatic HIV-1 infection: a randomised controlled trial. ANRS 048 study group. *Lancet*  1999; 353: 1923.
12. Bernstein ZP, Porter MM, Gould M, Lipman B, Bluman EM, Stewart CC et al. Prolonged administration of low-dose interleukin-2 in human immunodeficiency virus-associated malignancy results in selective expansion of innate immune effectors without significant clinical toxicity. *Blood* 1995; 86: 3287.
13. Jacobson EL, Pilaro F, Smith KS. Rationale interleukin 2 therapy for HIV positive individuals: daily low doses enhance immune function without toxicity. *Proc Natl Acad Sci. USA* 1996; 93: 1045.
14. Barouch DH, Craiu A, Kuroda MJ, et al. Augmentation of immune responses to HIV-1 and simian immunodeficiency virus DNA vaccines by IL-2/Ig plasmid administration in rhesus monkeys. *Proc Natl Acad Sci USA,* 2000; 97:4192.
15. Miró JM, Antela A, Arrizabalaga J, Clotet B, Gatell JM, Guerra L, Iribarren JA, Laguna F, Moreno S, Parras F, Rubio R, Santamaría JM, Viciana P por el Grupo de Estudio de Sida (GESIDA) y por Consejo Asesor Clínico (CAC) de la Secretaría Plan Nacional sobre el Sida (SPNS) del Ministerio de Sanidad y Consumo (MSC). Recomendaciones de GESIDA/Plan Nacional sobre el Sida respecto al tratamiento antirretroviral en pacientes adultos infectados por el vih en el año 2000*. www.gesidaseimc.com*. Edición de junio del 2000.

***

1. The first cycle is from week 0 to week 10, starting with two weeks without treatment. Viremia determinations in weeks 9, 19, 29 and 39 will be taken when it is possible to return to treatment in the following cycle, only patients with viremia >50 copies/mL in week 9 will stop treatment in week 10, etc. [↑](#footnote-ref-2)
2. Determinations of A2 and B25: only patients with both antigens (75% of the population) can take part; the presence of A2 or B25 is a requirement to measure the gag-specific cytotoxic response. [↑](#footnote-ref-3)
3. In patients with HLA35 or A2 (70% of the patients). [↑](#footnote-ref-4)
4. Amygdala biopsy. [↑](#footnote-ref-5)
